# NodeGWAS: Leveraging graph pangenomes for sensitive and accurate association analyses across diverse diploid and polyploid species

**DOI:** 10.1016/j.xplc.2026.101835

**Published:** 2026-03-25

**Authors:** Yixing Zhang, Yuheng Wang, Tingting Wu, Yan Lin, Yue Tan, Yiying Qi, Yuhao Wang, Baiyu Wang, Zhengguang Wang, Qing Zhang, Jisen Zhang, Yumin Huang, Haibao Tang

**Affiliations:** 1 Fujian Provincial Key Laboratory of Haixia Plant Systems Biology, Haixia Institute of Science and Technology & College of Life Science, Fujian Agriculture and Forestry University, Fuzhou 350002, China; 2 State Key Lab for Conservation and Utilization of Subtropical Agro-Biological Resources, Guangxi Key Lab for Sugarcane Biology, College of Agriculture, Guangxi University, Nanning 530004, China

Dear Editor,

This study introduces NodeGWAS, a graph pangenome-based framework that performs association analysis using graph nodes. Compared with conventional GWAS approaches, NodeGWAS strikes a balance between sensitivity and interpretability, and its applications in *Arabidopsis thaliana* and sugarcane demonstrate its effectiveness in dissecting complex traits.

Genome-wide association studies (GWAS) are essential for linking genomic variants to phenotypic traits (Tam et al., 2019). However, plant genomes vary widely in size, ploidy, heterozygosity, and repeat content (35%–90%), posing substantial challenges for genotyping (Wang et al., 2018). *k*-mer-based GWAS, which is reference-free, can detect a broader spectrum of variants beyond SNPs and insertions or deletions (Karikari et al., 2023) and has demonstrated strong performance compared with conventional approaches (Rahman et al., 2018; Voichek and Weigel, 2020) (Supplemental Table 1). However, it faces challenges in accurately mapping very short sequences to reference genomes and requires substantial computational resources (Supplemental Note 1).

In recent years, graph-based pangenomes have emerged as powerful alternatives to the standard linear reference genome, improving mapping accuracy and thereby enhancing downstream genotyping of both small variants and larger structural variants (Hickey et al., 2024). Nevertheless, studies on graph-based variant calling and genotyping remain limited (Supplemental Figure 1; Supplemental Table 2; Supplemental Note 2), whereas analytical approaches that directly leverage pangenome graph nodes hold considerable promise.

In this study, we propose NodeGWAS, a novel genotyping framework that operates directly on graph pangenomes and fills a critical gap in applying GWAS to species with high genetic diversity or complex structural variation—both of which pose substantial mappability challenges. In a graph pangenome, each node represents a non-redundant, variable-length “DNA word” (i.e., variable-length *k*-mers) that captures sequence variation across multiple genomes (Figure 1A; Supplemental Figures 2 and 3; Supplemental Table 2; Supplemental Note 3).

**Figure 1:**
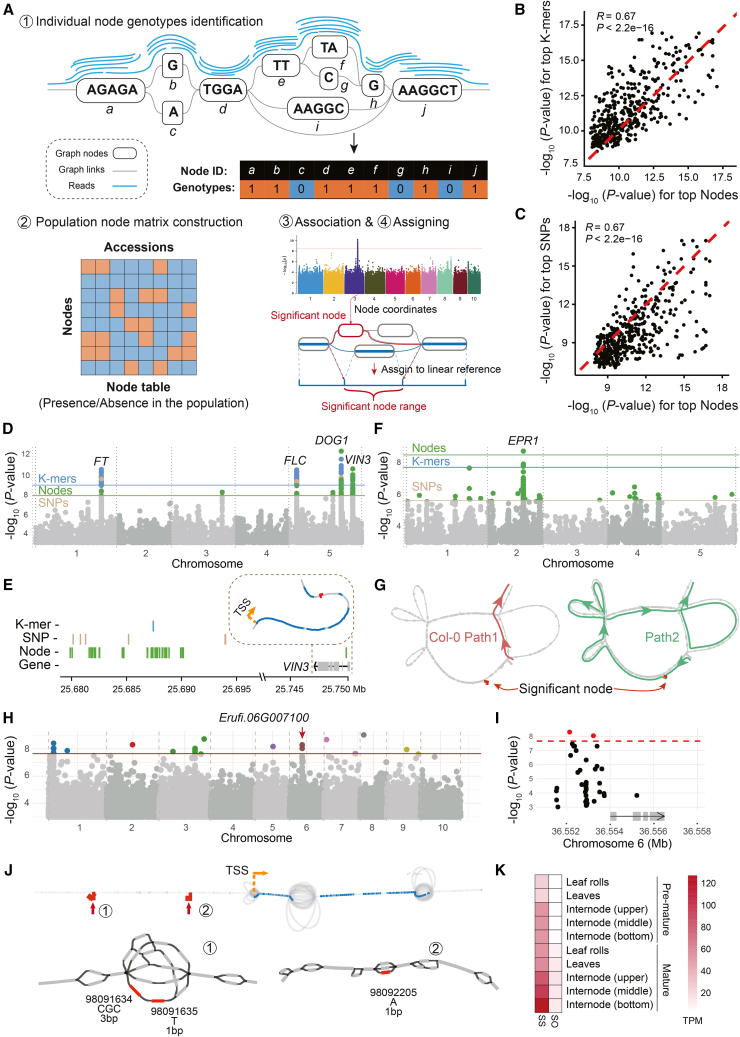
NodeGWAS: Workflow, performance evaluation, and application. **(A)** NodeGWAS workflow. **(1)** Individual node genotyping: Resequencing reads are aligned to the pangenome graph to determine the presence (1, orange) or absence (0, blue) of nodes (e.g., node *a* = “AGAGA”). **(2)** Matrix construction: Node genotypes from all individuals are compiled into a population-level matrix. **(3)** Association analysis: GWAS identifies nodes significantly associated with the trait. **(4)** Node assignment: Significant nodes are projected onto the linear reference genome to facilitate candidate gene discovery. **(B and C)** Correlation of top *p* values between top nodes and *k*-mers **(B)** or SNPs **(C)**. Pearson correlation coefficients (*R*) and corresponding *p* values were calculated using *t*-tests. **(D)** Manhattan plots of the flowering-time trait using three different methods. Only significant *k*-mers and SNPs are shown. *FLOWERING LOCUS T* (*FT), FLOWERING LOCUS C (FLC), DELAY OF GERMINATION 1* (*DOG1), and VERNALIZATION INSENSITIVE 3* (*VIN3)* are genes from *A. thaliana*. **(E)** Genomic positions of significant markers from three GWAS approaches. A graph pangenome representation of the *AT5G57380* locus and its flanking regions is shown. NodeGWAS identifies a significant node (red dot) within an intronic region. The orange arrow indicates the transcription start site (TSS), and coding sequences are shown in blue. **(F)** Manhattan plots of the root-length trait analyzed using three methods. Only significant *k*-mers and SNPs are shown. *EARLY PHYTOCHROME RESPONSE 1 (EPR1)* is a gene from *A. thaliana*. **(G)** Allelic paths near *EPR1* in the wild-type Col-0 and an alternative allele path (path 2), which contains the significant node (Node 34358731; red dot). **(H)** Manhattan plots of sucrose-content traits in sugarcane generated by NodeGWAS. A red arrow indicates significant signals near the *Erufi.06G007100* locus. **(I)** Zoomed-in Manhattan plot of a candidate region on chromosome 6, showing multiple significant loci in the TSS region of *Erufi.06G007100*. **(J)** Graph pangenome representation of the *Erufi.06G007100* locus and its flanking regions. Top: subgraph visualization with significant nodes indicated by red arrows. The orange arrow marks the TSS, and coding sequences are shown in blue. Bottom: enlarged view of significant nodes (red) and their flanking nodes. Significant nodes are labeled by node ID/sequence/length (e.g., 98091634/CGC/3 bp, 98091635/T/1 bp, and 98092205/A/1 bp). **(K)** Expression heatmap of *Erufi.06G007100* in *Saccharum spontaneum* (Ss) and *Saccharum officinarum* (So). Gene expression levels are shown as TPM (transcripts per million), normalized for gene length and sequencing depth.

To comprehensively evaluate NodeGWAS, we applied it to a classic *Arabidopsis thaliana* population phenotype dataset, which includes 1,487 traits and 1,047 individuals (Rahman et al., 2018; Voichek and Weigel, 2020), and benchmarked it against both linear reference GWAS and *k*-mer-based approaches. Although the *A. thaliana* genome is relatively simple, it provides a suitable test case before extending NodeGWAS to more genetically complex species (Supplemental Figure 4; Supplemental Note 4).

A total of 775 traits with significant associations were identified across the three methods, of which 416 were consistently detected by all approaches, indicating substantial overlap (Supplemental Figure 5). Notably, NodeGWAS identified an additional 51 traits with significant associations that were not detected by the other methods. Using 16 randomly selected resequencing datasets, we further observed that, based on the graph pangenome, the alignment score increased from 96.98 to 99.00, and the mapping rate improved from 79.20% to 81.24%. The overlap of significant loci among the three methods was limited, and the number of regions uniquely identified by NodeGWAS fell between those identified by the *k*-mer- and SNP-based approaches (Supplemental Figure 6). In terms of sensitivity and statistical significance, the number of significant variants and the top *pp* values were highly correlated across methods (*R* = 0.67–0.87; Figures 1B and 1C; Supplemental Figures 7A and 7B). NodeGWAS outperformed the SNP-based method but showed lower sensitivity than the *k*-mer-based approach (Supplemental Note 5). Although *k*-mer-based methods exhibit higher apparent sensitivity to GWAS signals, our results indicate that NodeGWAS provides superior control of false positives compared with unstructured *k*-mer approaches (Supplemental Figures 8A and 8B; Supplemental Note 6).

It is worth noting that, due to the inherent properties of *k*-mers, a single variant may generate dozens or even hundreds of *k*-mers, leading to substantial redundancy and potential over-reporting. In contrast, NodeGWAS reports only polymorphic nodes that are in linkage disequilibrium. *k*-merGWAS identified 776,634 significant *k*-mers across 1,487 traits, of which 33.7% could not be reliably assigned to the reference genome (Supplemental Figure 9). By contrast, NodeGWAS leverages graph structure and node context (e.g., the link field in graphical fragment assembly (GFA); Supplemental Figure 2; Supplemental Table 2) to map all 87,510 significant nodes to reference coordinates, whereas the conventional SNP-based method identified only 28,954 loci. Overall, NodeGWAS achieves a balance between sensitivity and interpretability.

We further evaluated NodeGWAS using the canonical flowering time trait (10°C) in *A. thaliana* and successfully recapitulated all well-established loci, including *Flowering Locus T*, *Short Vegetative Phase*, *Flowering Locus C*, *Delay of Germination 1*, and *Vernalization Insensitive 3* (Figure 1D). Additionally, NodeGWAS identified two signal intervals that were not detected by either the linear reference or *k*-mer-based approaches. Near the *VIN3* locus, NodeGWAS identified 55 significant nodes. In comparison, *k*-merGWAS detected only two significant *k*-mers, with just one mapping to chromosome 5 and the other remaining unmapped. SNP-based GWAS identified five significantly associated SNPs in the downstream regulatory region of *VIN3*. Notably, one of the significant nodes was located within an intron of *VIN3*, suggesting a novel causal variant that may influence gene function (Figure 1E).

NodeGWAS revealed distinct advantages for detecting certain traits. For instance, we identified novel significant loci near *Early Phytochrome Response 1* associated with root length responses to iron availability, which were not detected by *k*-mer- or SNP-based GWAS (Figure 1F; Supplemental Figure 10; Satbhai et al., 2017; Kumari et al., 2019). Iron may regulate phytochrome function by influencing the cellular redox state or modulating iron–sulfur clusters, thereby affecting root growth (Rockwell and Lagarias, 2020). By examining the positions of significant nodes, we identified 30 distinct paths among the 97 samples (Supplemental Figures 2 and 11; Supplemental Table 2; Supplemental Note 7). Notably, eight samples followed path 2, which contains a large insertion relative to path 1 (Figure 1G). The presence of the significant node within path 2 is associated with increased root length. This node is located within a small bubble inside the inserted fragment (highlighted in red in Supplemental Figure 12), which may complicate the detection of large structural variants and lead to failure to detect these loci. This observation provides empirical support for the ‘nested variation’ concept described above. Additionally, NodeGWAS demonstrated improved control of false positives for certain traits (Supplemental Figure 13; Supplemental Note 8).

Compared with the small, relatively simple diploid genome of *A. thaliana*, genotyping complex polyploids has long been plagued by high error rates and difficulties in comparing individuals across varying ploidy levels, severely limiting its downstream applications in population genetics (Supplemental Figure 14; Phillips, 2024). By introducing a graph-based pangenome reference, NodeGWAS mitigates the alignment bias associated with a single reference, captures comprehensive genetic diversity, and effectively resolves alignment challenges in polyploids. It also sidesteps common errors inherent to traditional polyploid genotyping while maintaining the accuracy and completeness of genetic information by using node coverage/counts as GWAS predictors. Consequently, NodeGWAS is naturally adaptable to species with varying ploidy levels.

To illustrate this advantage, we applied NodeGWAS to sugarcane, a modern cultivated crop with a highly polyploid genome. This genome is derived from interspecific hybridization among multiple *Saccharum* species or varieties and is among the most complex and diverse crop genomes (Zhang et al., 2025). Owing to its variable ploidy levels and hybrid composition, accurate genotyping in sugarcane remains a considerable challenge. We performed permutation tests to ensure rigorous false-positive control and fair comparisons (Supplemental methods). Across eight sugar-related traits, SNP-based GWAS yielded no significant associations, whereas *k*-mer-based and NodeGWAS approaches identified 4,751 and 544 loci, respectively (Supplemental Figure 15). Statistical power followed the order *k*-mer > node > SNP (Supplemental Figure 16). The limited overlap between *k*-mer and NodeGWAS results (e.g., only nine shared quantitative trait loci for sucrose content) indicates that NodeGWAS provides a complementary approach by capturing distinct genomic signals (Supplemental Table 3). Compared with previous allele-level linear reference studies (Zhang et al., 2025), NodeGWAS identified more significant loci and yielded stronger statistical signals (Supplemental Figure 17). In total, NodeGWAS detected 143 loci across more than half of the traits. For example, at ∼36 Mb on chromosome 6, we identified three nodes significantly associated with four of the eight traits (Figure 1H; Supplemental Figure 18A). The presence of these nodes significantly increases the four sugar-related traits (Supplemental Figures 18B and 18C), and they are predominantly found in *Saccharum officinarum* (Supplemental Figure 18D), which contributes high sugar content to modern cultivars. Using a pangenome subgraph and coordinate conversion to a linear reference genome, we determined that these three loci reside in the promoter region of *Erufi.06G007100* (Figures 1I and 1J), which encodes a glucuronosyltransferase and is highly homologous to *IRREGULAR XYLEM 10*. Transcriptome analysis further showed that *Erufi.06G007100* is consistently expressed at lower levels in stems and leaves of *S. officinarum* across developmental stages compared with *Saccharum spontaneum* (Figure 1K), implicating a regulatory role in sugar accumulation.

In this study, we present NodeGWAS, a novel strategy for performing GWAS directly on graph-based genotypes defined by structurally resolved nodes. Using this end-to-end workflow, we demonstrate that NodeGWAS reduces redundancy and false positives and uncovers novel candidate genes in *A. thaliana* that are missed by traditional approaches. We further extend NodeGWAS to polyploid sugarcane, where it identifies highly significant functional loci associated with sugar-content traits that would otherwise remain undetected. Overall, our work provides a robust and interpretable framework applicable to both diploid and polyploid genomes (Supplemental Note 9).

## Data and code availability

The code and full documentation are available on GitHub: https://github.com/zhangyixing3/NodeGWAS.

## Funding

This work was supported by the 10.13039/501100012166National Key Research and Development Program (2024YFF1000800 to H.T. and J.Z.) and the 10.13039/501100001809National Natural Science Foundation of China (grant 32500548 to Y.H.).

## Acknowledgments

No conflict of interest is declared.

## Author contributions

H.T., Y.H., and J.Z. conceived and designed the project. Y.Z. performed the main analyses, drafted the initial manuscript, and implemented the pipeline in Rust. Yuheng Wang, T.W., Y.L., Y.T., Y.Q., Yuhao Wang, B.W., and Z.W. contributed to data analysis and pipeline evaluation. H.T., Y.H., J.Z., and Q.Z. revised the manuscript. All authors read and approved the final version.

## Supplemental information

Document S1. Supplemental Figures 1–18, Supplemental Tables 1–4, Supplemental Notes 1–9, and supplemental methods

Document S2. Article plus supplemental information
