# Supplementary material for: NodeGWAS: Leveraging graph pangenomes for sensitive and accurate association analyses across diverse diploid and polyploid species
Source: Plant Commun. 2026 Mar 25;7(7):101835. doi: 10.1016/j.xplc.2026.101835 (PMC13370226; doi:10.1016/j.xplc.2026.101835)
Supplement: Document S1. Supplemental Figures 1–18, Supplemental Tables 1–4, Supplemental Notes 1–9, and supplemental methods [file mmc1.pdf]

Plant Communications, Volume 7

Supplemental information

NodeGWAS: Leveraging graph pangenomes for sensitive and accurate association analyses across diverse diploid and polyploid species

Yixing Zhang, Yuheng Wang, Tingting Wu, Yan Lin, Yue Tan, Yiying Qi, Yuhao Wang, Baiyu Wang, Zhengguang Wang, Qing Zhang, Jisen Zhang, Yumin Huang, and Haibao Tang

NodeGWAS: Leveraging graph pangenomes for sensitive and accurate association analysis across diverse diploid and polyploid species

Yixing Zhang[1], Yuheng Wang[1], Tingting Wu[1], Yan Lin[1], Yue Tan[1], Yiying Qi[1], Yuhao Wang[2], Baiyu Wang[2], Zhengguang Wang[1], Qing Zhang[2], Jisen Zhang[2,*], Yumin Huang[1,*], Haibao Tang[1,*]

[1.] Fujian Provincial Key Laboratory of Haixia Plant Systems Biology, Haixia Institute of Science and Technology & College of Life Science, Fujian Agriculture and Forestry University, Fuzhou, 350002 China.
[2.] State Key Lab for Conservation and Utilization of Subtropical Agro-Biological Resources, Guangxi Key Lab for Sugarcane Biology, College of Agriculture, Guangxi University, Nanning 530004, China.

* To whom correspondence may be addressed. Email: zjisen@126.com, ymhuang@fafu.edu.cn or tanghaibao@gmail.com

Supplemental Methods

Pangenome construction

69 *A. thaliana* accessions were downloaded from NCBI (NCBI BioProject accession: PRJNA1033522) (Lian et al. 2024). 32 *A. thaliana* accessions were downloaded from Figshare (https://figshare.com/articles/dataset/32_ecotypes_Arabidopsis_thaliana_genomes_gene_annotation_pan-TE_library_graph_pan-genome_gene_family_and_gene_presence_absence_matrices_files_/21673895) (Kang et al. 2023). The T2T *A. thaliana* genome was downloaded from GitHub (https://github.com/schatzlab/Col-CEN) (Naish et al. 2021).

The graph-based pangenome of *A. thaliana* was constructed using 97 non-redundant genome assemblies through the Minigraph-Cactus pipeline (version 5.12.0) under parameters of "--reference 035_Col_CEN_v12 --filter 10 --vcf full clip filter --giraffe --gfa full clip filter --gbz full clip filter --draw full clip --viz full clip --odgi full clip filter", with the telomere-to-telomere (T2T) *A. thaliana* genome serving as the reference. To align the resequencing data, the GFA graph, which was filtered by removing nodes covered by fewer than 10 haplotypes, was used.

Using the PGGB pipeline (version 0.5.2), we constructed a pangenome graph incorporating the AP85-441, LA-purple, Np-X, ZG, XTT22, R570 and *Erianthus rufipilus* genomes, which was subsequently decomposed into VCF files (Zhang et al. 2025; Q. Zhang et al. 2022; Zhang et al. 2018; Wang et al. 2023; Healey et al. 2024). These VCF files were then merged with VCF data from 60 *E. rufipilus* accessions, and a comprehensive pangenome was constructed using the *vg autoindex* tool with default parameters based on the linear reference genome of *E. rufipilus* (Garrison et al. 2018; Wang et al. 2023; Huang et al. 2026).

Data alignment

Resequencing data for 1,047 *A. thaliana* accessions were obtained from the NCBI Sequence Read Archive (SRA accession: SRP056687) (1001 Genomes Consortium. Electronic address: magnus.nordborg@gmi.oeaw.ac.at and 1001 Genomes Consortium 2016). For sugarcane, whole-genome resequencing data for 183 accessions were downloaded from the SRA (Accessions: SRR29827487-SRR29827669).

The resequencing reads were aligned to the pangenome graph using *vg giraffe* (version v1.61.0) with default settings (Sirén et al. 2021). The mapping results were filtered using *vg filter* with the parameters: -r 0.90 -fu -m 1 -q 15 -D 999. The filtration criteria were specifically devised to retain high-quality alignments with mapping quality exceeding 15, a primary alignment score of at least 0.90, and at least one match at each terminus, enabling the trimming of reads.

Computational resources and benchmarking

To demonstrate the practical efficiency of our framework, we directly compared the computational demands of NodeGWAS and GATK (using the "-ploidy 4" mode) on a real tetraploid potato population dataset (F. Zhang et al. 2022). All analyses were performed on a computing node equipped with 128 CPU cores and 512 GB RAM. The results show that GATK required approximately 51.9 hours (3,114 minutes) to process a single sample, whereas NodeGWAS completed the same task in only 6.2 hours (372 minutes), an approximately 8.36-fold faster performance (Supplemental Table 4). Peak memory usage of NodeGWAS (26.79 GB) was comparable to GATK (30.99 GB) in polyploid mode and remained within highly accessible ranges (Supplemental Table 4).

Permutation testing for k-mers, SNPs, and nodes in A. thaliana

Like *k*-mer-based GWAS, the EMMA (emma.REMLE function, emma.R) was employed to estimate the variance components, which were subsequently used to compute the phenotypic covariance matrix (Kang et al. 2008). We then performed 100 permutations of the phenotype using the mvnpermute R package (version 1.0.1) (Abney 2015). The family-wise error rate (FWER) threshold at *n*% (for instance, *n*=5 corresponds to a 5% threshold) was determined by selecting the *n*-th top *P* value from the 100 top *P* values obtained from performing genome-wide association analysis on each permutation.

Genome-wide association analysis of A. thaliana

In NodeGWAS, each alignment file was converted to JSON format by *vg view*, which enabled us to directly process the alignment information for each read and count the occurrences of nodes that are perfectly matched by the reads. Nodes with a count of two or more were considered as "presence", while counts fewer than two were considered as "absence". Finally, we combined the presence/absence information for all nodes from all accessions and generated a presence/absence matrix. The status of presence/absence of nodes was converted to biallelic genotypes for association. Nodes with minor allele frequency (MAF) < 0.05 were filtered. GWAS was performed by LMMs with the kinship matrix in GEMMA (version 0.98.5) (Zhou and Stephens 2012).

A. thaliana GWAS results including *k*-mer and SNP analyses were downloaded from Zenodo (https://zenodo.org/records/3701176#.XmX9u5NKhhE) (Voichek and Weigel 2020).

Genome-wide association analysis of Saccharum spp.

Like *A. thaliana*, we obtained the filtered genotype files. GWAS was performed by Mixed-Model Association eXpedited program (EMMAX, version 20120210) (Kang et al. 2010), with the first ten principal components (PCs) included as a random effect matrix. The genome-wide significance threshold was determined by Bonferroni correction and was set to 2.19068e-08. We also evaluated the performance between NodeGWAS and SNP, *k*-mer-based GWAS. To ensure rigorous false-positive control and establish fair significance thresholds, we performed 100 permutation tests by randomly shuffling phenotypes while

keeping genotype and kinship matrices constant. The 5th percentile of the minimum *P* values from permutations defines the significant threshold.

Conversion of genomic coordinates

For each trait, we extracted the genomic regions within ±5000 bp of the significantly associated loci using bedtools (version 2.30.0) and merged overlapping regions with bedtools merge to obtain non-redundant genomic intervals (Quinlan and Hall 2010). To further compare the distribution patterns of associated loci identified by the *k*-merGWAS, NodeGWAS and SNP-based methods, we employed bedtools intersect to quantify the overlapping regions among different methods and bedtools subtract to identify method-specific genomic regions.

Inject back into the linear genome

For nodes located on the linear reference genome (e.g., the T2T Col *Arabidopsis* reference genome), we determine their coordinates by calculating the cumulative length offset of each node along the reference sequence. For nodes that are not part of the linear reference genome (such as those formed by insertions or other variants), we locate the genomic position at which the variant represented by the node is inserted/deleted into the reference genome and use that position as the node's coordinate.

Supplementary Notes

1. Limitations of k-mer-based GWAS

Accurately linking extremely short fixed-length *k*-mer sequences (e.g., 31 bp) to specific genomic regions in plant reference genomes remains a significant challenge, particularly in species with high ploidy levels or abundant repetitive sequences. For example, using *k*-merGWAS, only 1,542 (5.4%) and 3,929 (3.6%) significant *k*-mers could be directly and accurately mapped to the reference genome, for maize kernel oil content and leaf angle trait, respectively (He et al. 2024). On the other hand, compared to traditional genetic markers, *k*-mer analysis requires substantially more computational resources (Karikari et al. 2023). An example is the detection of 1.1 billion *k*-mers from 261 inbred lines, with the majority of lines containing 0.35–0.55 billion *k*-mers (He et al. 2024). Such a large volume and complexity of data presents considerable challenges for computation.

2. Challenges in graph-based genotyping

Traditional graph-based genotyping methods (such as PanGenie, *vg call*, etc.) (Hickey et al. 2020; Ebler et al. 2022) are primarily optimized for diploid genomes, which are fundamentally incompatible with the complex allele dosage effects in polyploids. Node-based analysis "sidesteps" this by using graph node coverage/counts as predictors (Supplemental Figure 1). This makes it naturally adaptable to species with varying ploidy levels. Meanwhile, large structural variants (SVs) are particularly challenging, as they often form complex, nested 'bubble' structures (Supplemental Table 2) that are both difficult to detect and also poorly represented in the VCF format (Romain et al. 2025). Node-based analysis avoids this by decomposing complex topologies into their most basic units—nodes. It does not need to decide whether a complex region is "one SV" versus "multiple SVs," nor does it require stable breakpoints in a VCF representation (Supplemental Figure 1). Finally, traditional graph-based variant calling typically surjects read alignments (Supplemental Table 2) onto a chosen linear reference genome and then is used with conventional linear-based variant callers. Node-based analysis bypasses the severe information loss inherent to traditional linear-based SV calling pipelines. Furthermore, this two-stage workflow is cumbersome, as it requires handling SNPs and SVs through separate pipelines.

3. NodeGWAS workflow

In a sequence-resolved variation graph, any sequence-level variation (including SNPs, INDELs and complex SVs) triggers the creation of distinct, alternative nodes, and is encoded within the topology of the graph. NodeGWAS treats each node as an independent genetic unit. The 0/1 genotype matrix successfully distinguishes these variants and captures their distinct biological implications (Supplemental Figure 3). Thus, our node-based association analysis preserves the full spectrum of genomic variation. This "node-as-a-feature" approach allows NodeGWAS to perform association mapping across the entire genomic spectrum without the need for explicit SV calling, thereby bypassing reference bias and complexity constraints. NodeGWAS follows a four-stage workflow

(Figure 1A): (1) Align each individual resequencing data to the graph pangenome to identify the genotypes (presence or absence) of each node, (2) Collate the node genotypes of all individuals to build a population-level node matrix (3) Perform GWAS analysis using node matrix, with appropriate covariate and population-structure controls, to identify significantly associated nodes. (4) Assign node coordinates back to the linear reference genome for downstream annotation. NodeGWAS is agnostic of the graph pangenome construction methods and can directly work with prebuilt pan-genomes constructed with Minigraph-Cactus, PGGB, or Vg (Hickey et al. 2024; Garrison et al. 2024, 2018).

4. A. thaliana graph pangenome

We first constructed a high-quality *A. thaliana* graph pangenome using the Minigraph-Cactus pipeline, which incorporates 97 diverse assemblies from previous studies (Lian et al. 2024; Kang et al. 2023). The growth curve, generated using Panacus to model the relationship between the number of genomes included and the total graph size, indicated that the pangenome has reached saturation at 97 genomes (Supplemental Figure 4) (Parmigiani et al. 2024). This suggests that the graph-based pangenome has effectively captured the genetic diversity of the Arabidopsis population and can serve as a comprehensive reference for downstream analyses.

5. Comparison of GWAS methods in significant loci detection

Compared to the SNP-based approach, NodeGWAS identified more significant loci in 27.3% of the traits, whereas the SNP-based method did so in only 16.6%. In comparison to the *k*-mer-based approach, NodeGWAS detected fewer significant loci in only 32.9% of the traits (*k*-mer-based: 12.4%). In terms of association strength, NodeGWAS exceeded the top-hit signals of the SNP-based method in 29.4% of the traits, with the remaining (15.8%) showing lower significance. Similarly, compared to the *k*-mer-based method, NodeGWAS demonstrated stronger signals in 15.2% of the traits (*k*-mer-based: 29.4%).

6. False Positive Assessment via Permutation Tests

To evaluate the accuracy across different methods in *A. thaliana*, we randomly selected 125 traits and performed 100 phenotype permutations for each, while maintaining the original genotype matrix and covariates.

The Mean number of False Positives (MFP) is defined as the total number of significant loci observed across 100 permutation tests divided by 100:

$$\mathrm{FPR} = \frac{\sum_{i=1}^{100} N_i}{100}$$

where N_i is the number of significant loci detected in the i-th permutation.

we observed that the *k*-mer-based method suffers from the highest error rate, followed by Node, with SNP the lowest (Supplemental Figure 8A, Wilcoxon test, *P* < 0.001). The SNP- and Node-based methods have similarly low Mean number of False Positives (MFP) with

no significant difference, whereas the *k*-mer-based method exhibits a significantly higher MFP than the others (Supplemental Figure 8B, Wilcoxon test, $P < 0.001$).

7. paths near the significant nodes

The total length ranges from 125 to 1,956 bp. These paths are not structurally independent, as multiple paths often share common subsequences, which are represented as shared nodes in the graph (Supplemental Figure 11).

8. A. thaliana quantitative disease resistance trait

For the quantitative disease resistance trait (Debieu et al. 2016), the number of markers remaining after quality control using the *k*-merGWAS, SNP analysis, and NodeGWAS was 170,382,079, 793,248 and 10,518,414, respectively; of these, the number of significant associations called by each method was 4,750, 115, and 49,023, respectively. Although all three approaches pinpointed the same 16.7Mb region on chromosome 5, *k*-merGWAS exhibited a high false-positive rate, consistent with its tendencies to over-report (Supplemental Figure 13) - whereas NodeGWAS produced a sharp, well-defined peak capturing only the nodes in LD, without spurious signals.

9. Discussion of NodeGWAS

Unlike *vg call* - which relies on explicit detection and VCF representation of complex structural variants manifesting as nested bubbles (Supplemental Figure 2 and Supplemental Table 2) (Paten et al. 2018, 2017; Garrison et al. 2018, 2022) - NodeGWAS genotypes nodes directly, sidestepping the challenge of SV calling and representation (Romain et al. 2025). Secondly, in contrast to *vg inject*, our method is more efficient and streamlined. *vg inject* requires injecting alignments from BAM files back to the linear reference genome and subsequent separation of SNP and SV discovery. Third, compared to *k*-mer-based GWAS, our method leverages the link field (*L*) in the GFA file to precisely locate variants on the reference genome. *k*-mer-based approaches often over-report loci with substantial redundancy, which are difficult to map and interpret, computationally expensive, and, although seemingly more sensitive, have been shown to significantly increase false positive rates. Fourth, the pangenome can comprehensively capture the genetic diversity of a species and effectively reduce analytical bias caused by reliance on a single reference genome.

Despite its ability to alleviate reference bias through direct graph-based analysis, NodeGWAS is still constrained by several limitations. First, computational scalability remains a challenge for ultra-large polyploid genomes, as the requirements for graph construction, mapping and node-counting increase significantly. Second, highly repetitive sequences may complicate graph topology, potentially leading to non-specific node mapping and reduced statistical power in these regions, though we note that this limitation is shared with linear-reference approaches. Finally, the comprehensiveness of the graph is inherently limited by the quality and representative diversity of the input genomes. Incomplete or biased sampling of founder genomes may reduce sensitivity for rare or population-specific variation. As more high-quality, telomere-to-telomere (T2T) assemblies

are getting integrated into pan-genome frameworks, the resolution and discovery power of NodeGWAS will continue to improve.

1001 Genomes Consortium. Electronic address: magnus.nordborg@gmi.oeaw.ac.at, and 1001 Genomes Consortium. 2016. “1,135 Genomes Reveal the Global Pattern of Polymorphism in Arabidopsis Thaliana.” *Cell* 166 (2): 481–491.

Abney, Mark. 2015. “Permutation Testing in the Presence of Polygenic Variation.” *Genetic Epidemiology* 39 (4): 249–258.

Debieu, Marilyne, Carine Huard-Chauveau, Anne Genissel, Fabrice Roux, and Dominique Roby. 2016. “Quantitative Disease Resistance to the Bacterial Pathogen Xanthomonas Campestris Involves an Arabidopsis Immune Receptor Pair and a Gene of Unknown Function.” *Molecular Plant Pathology* 17 (4): 510–520.

Ebler, Jana, Peter Ebert, Wayne E. Clarke, et al. 2022. “Pangenome-Based Genome Inference Allows Efficient and Accurate Genotyping across a Wide Spectrum of Variant Classes.” *Nature Genetics* 54 (4): 518–525.

Garrison, Erik, Andrea Guarracino, Simon Heumos, et al. 2024. “Building Pangenome Graphs.” *Nature Methods* 21 (11): 2008–2012.

Garrison, Erik, Zev N. Kronenberg, Eric T. Dawson, Brent S. Pedersen, and Pjotr Prins. 2022. “A Spectrum of Free Software Tools for Processing the VCF Variant Call Format: Vcflib, Bio-Vcf, cyvcf2, Hts-Nim and Slivar.” *PLoS Computational Biology* 18 (5): e1009123.

Garrison, Erik, Jouni Sirén, Adam M. Novak, et al. 2018. “Variation Graph Toolkit Improves Read Mapping by Representing Genetic Variation in the Reference.” *Nature Biotechnology* 36 (9): 875–879.

Healey, A. L., O. Garsmeur, J. T. Lovell, et al. 2024. “The Complex Polyploid Genome Architecture of Sugarcane.” Nature 628, 804–810.

He, Cheng, Jacob D. Washburn, Nathaniel Schleif, et al. 2024. “Trait Association and Prediction through Integrative K-Mer Analysis.” *The Plant Journal: For Cell and Molecular Biology* 120 (2): 833–850.

Hickey, Glenn, David Heller, Jean Monlong, et al. 2020. “Genotyping Structural Variants in Pangenome Graphs Using the vg Toolkit.” *Genome Biology* 21 (1): 35.

Hickey, Glenn, Jean Monlong, Jana Ebler, et al. 2024. “Pangenome Graph Construction from Genome Alignments with Minigraph-Cactus.” *Nature Biotechnology* 42 (4): 663–673.

Huang, Yumin, Yixing Zhang, Qing Zhang, et al. 2026. “Multiscale Pangenome Graphs Empower the Genomic Dissection of Mixed-Ploidy Sugarcane Species.” *Science (New York, N.Y.)* 391 (6785): eadx1616.

Kang, Hyun Min, Jae Hoon Sul, Susan K. Service, et al. 2010. “Variance Component Model to Account for Sample Structure in Genome-Wide Association Studies.” *Nature Genetics* 42 (4): 348–354.

Kang, Hyun Min, Noah A. Zaitlen, Claire M. Wade, et al. 2008. “Efficient Control of Population Structure in Model Organism Association Mapping.” *Genetics* 178 (3): 1709–1723.

Kang, Minghui, Haolin Wu, Huanhuan Liu, et al. 2023. “The Pan-Genome and Local Adaptation of Arabidopsis Thaliana.” *Nature Communications* 14 (1): 6259.

Karikari, Benjamin, Marc-André Lemay, and François Belzile. 2023. "K-Mer-Based Genome-Wide Association Studies in Plants: Advances, Challenges, and Perspectives." *Genes* 14 (7): 1439.

Lian, Qichao, Bruno Huettel, Birgit Walkemeier, et al. 2024. "A Pan-Genome of 69 Arabidopsis Thaliana Accessions Reveals a Conserved Genome Structure throughout the Global Species Range." *Nature Genetics* 56 (5): 982–991.

Naish, Matthew, Michael Alonge, Piotr Wlodzimierz, et al. 2021. "The Genetic and Epigenetic Landscape of the Arabidopsis Centromeres." *Science (New York, N.Y.)* 374 (6569): eabi7489.

Parmigiani, Luca, Erik Garrison, Jens Stoye, Tobias Marschall, and Daniel Doerr. 2024. "Panacus: Fast and Exact Pangenome Growth and Core Size Estimation." *Bioinformatics (Oxford, England)* 40 (12): btae720.

Paten, Benedict, Jordan M. Eizenga, Yohei M. Rosen, Adam M. Novak, Erik Garrison, and Glenn Hickey. 2018. "Superbubbles, Ultrabubbles, and Cacti." *Journal of Computational Biology: A Journal of Computational Molecular Cell Biology* 25 (7): 649–663.

Paten, Benedict, Adam M. Novak, Jordan M. Eizenga, and Erik Garrison. 2017. "Genome Graphs and the Evolution of Genome Inference." *Genome Research* 27 (5): 665–676.

Quinlan, Aaron R., and Ira M. Hall. 2010. "BEDTools: A Flexible Suite of Utilities for Comparing Genomic Features." *Bioinformatics (Oxford, England)* 26 (6): 841–842.

Romain, Sandra, Siegfried Dubois, Fabrice Legeai, and Claire Lemaitre. 2025. "Investigating the Topological Motifs of Inversions in Pangenome Graphs." In *bioRxiv*. March 17. https://doi.org/10.1101/2025.03.14.643331.

Sirén, Jouni, Jean Monlong, Xian Chang, et al. 2021. "Pangenomics Enables Genotyping of Known Structural Variants in 5202 Diverse Genomes." *Science* 374 (6574): abg8871.

Voichek, Yoav, and Detlef Weigel. 2020. "Identifying Genetic Variants Underlying Phenotypic Variation in Plants without Complete Genomes." *Nature Genetics* 52 (5): 534–540.

Wang, Tianyou, Baiyu Wang, Xiuting Hua, et al. 2023. "A Complete Gap-Free Diploid Genome in Saccharum Complex and the Genomic Footprints of Evolution in the Highly Polyploid Saccharum Genus." *Nature Plants* 9 (4): 554–571.

Zhang, Feng, Li Qu, Yincong Gu, Zhi-Hong Xu, and Hong-Wei Xue. 2022. "Resequencing and Genome-Wide Association Studies of Autotetraploid Potato." *Molecular Horticulture* 2 (1): 6.

Zhang, Jisen, Yiying Qi, Xiuting Hua, et al. 2025. "The Highly Allo-Autopolyploid Modern Sugarcane Genome and Very Recent Allopolyploidization in Saccharum." *Nature Genetics* 57 (1): 242–253.

Zhang, Jisen, Xingtan Zhang, Haibao Tang, et al. 2018. "Allele-Defined Genome of the Autopolyploid Sugarcane Saccharum Spontaneum L." *Nature Genetics* 50 (11): 1565–1573.

Zhang, Qing, Yiying Qi, Haoran Pan, et al. 2022. "Genomic Insights into the Recent Chromosome Reduction of Autopolyploid Sugarcane Saccharum Spontaneum." *Nature Genetics* 54 (6): 885–896.

Zhou, Xiang, and Matthew Stephens. 2012. “Genome-Wide Efficient Mixed-Model Analysis for Association Studies.” *Nature Genetics* 44 (7): 821–824.

Supplementary Figure

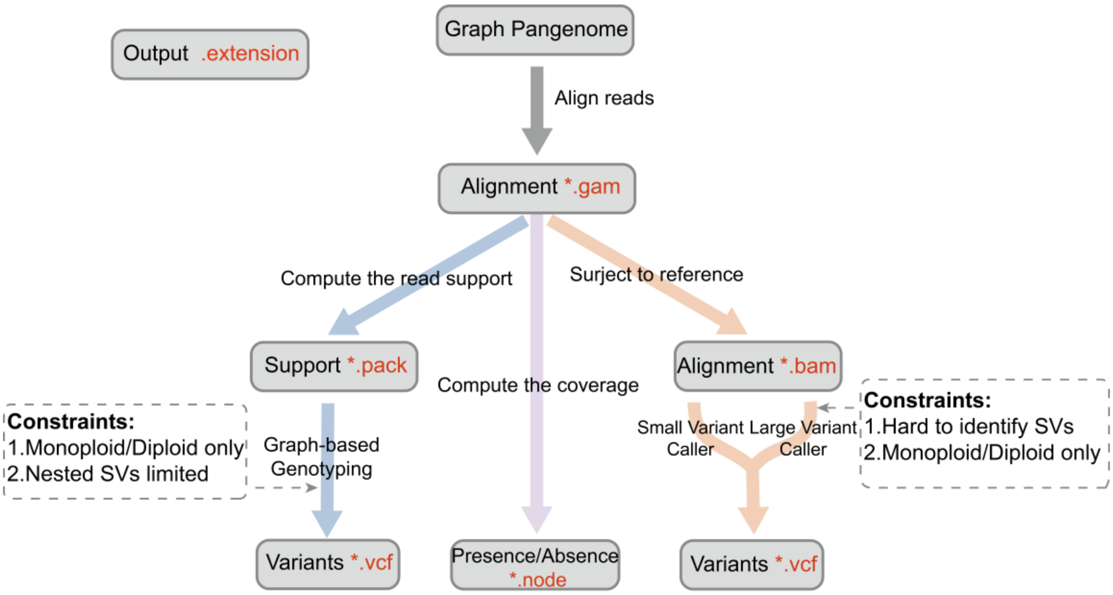

Supplemental Figure 1. Schematic of the GWAS workflow based on a graph pangenome. The workflow utilizes a graph pangenome as the reference. Following read alignment, an alignment file in .gam format is generated, which is then processed through three distinct analytical pathways: Left pathway (Graph-based genotyping): Genotyping is performed by computing read support (outputting .pack files) to ultimately generate variants. The constraints of this path include limited support for non-monoploid/diploid genomes and restricted capability in handling nested SVs. Middle pathway (Node-based analysis): This path directly computes the coverage of graph nodes to analyze Node presence/absence variations. Right pathway: Graph alignments are projected back onto a linear reference genome and converted into standard .bam files. Subsequently, small or large variant callers are employed to generate .vcf files. This approach faces challenges in de novo identifying SVs and is similarly constrained by organism polyploidy (supporting monoploid/diploid only).

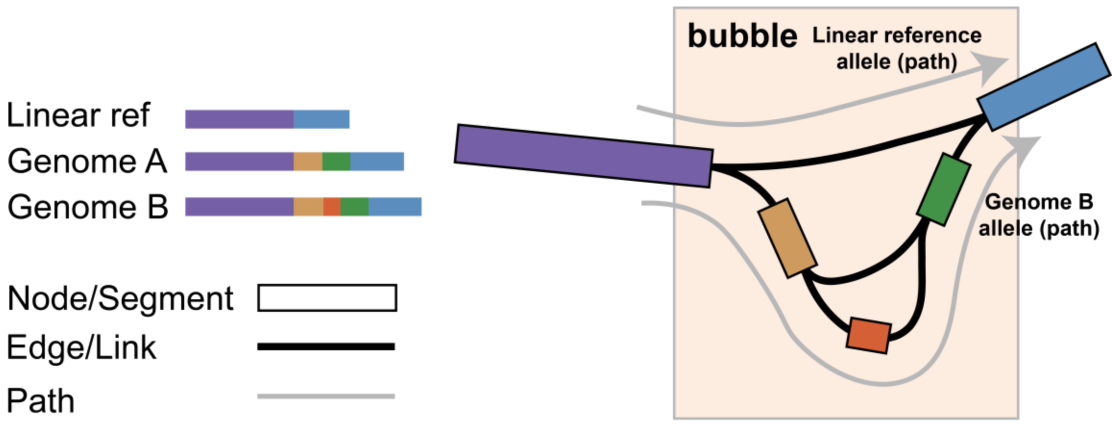

Supplemental Figure 2. Representation of a polymorphic locus in a pangenome graph takes the form of a ‘‘bubble’’ structure, which diverges at a source node where genomic sequences differ and converges at a sink node where sequences realign. Each distinct path from the source to the sink represents a specific allele or haplotype.

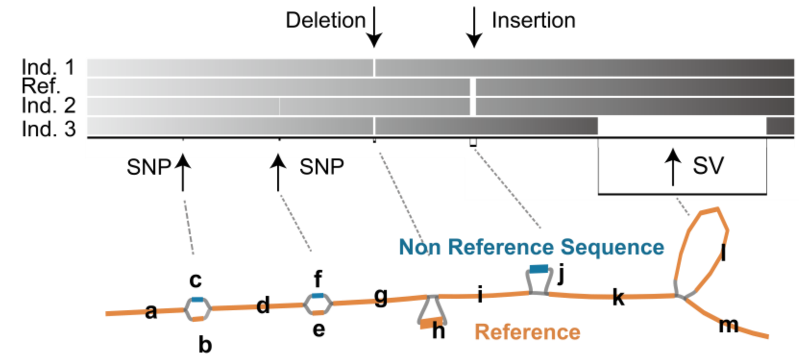

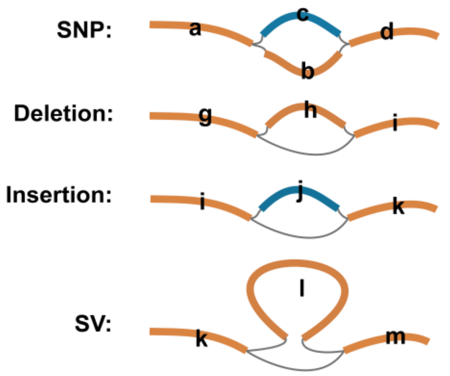

	Ind.1	Ind.2	Ind.3
a	1	1	1
b	1	1	0
c	0	0	1
d	1	1	1
e	1	1	0
f	0	0	1
g	1	1	1
h	0	1	0
i	1	1	1
j	1	0	1
k	1	1	1
l	1	1	0
m	1	1	1

Supplemental Figure 3. Illustration of the node-based variation representation in NodeGWAS. Four individual sequences (Ind. 1-3 and a Reference) containing two SNPs, one deletion, one insertion, and one structural variant (SV). Node "*h*" specifically captures a deletion event, while node "*l*" represents a large SV sequence. All variations within the pangenome can be represented by distinct nodes. By constructing a nodetable, we can effectively reconstruct the node-level information.

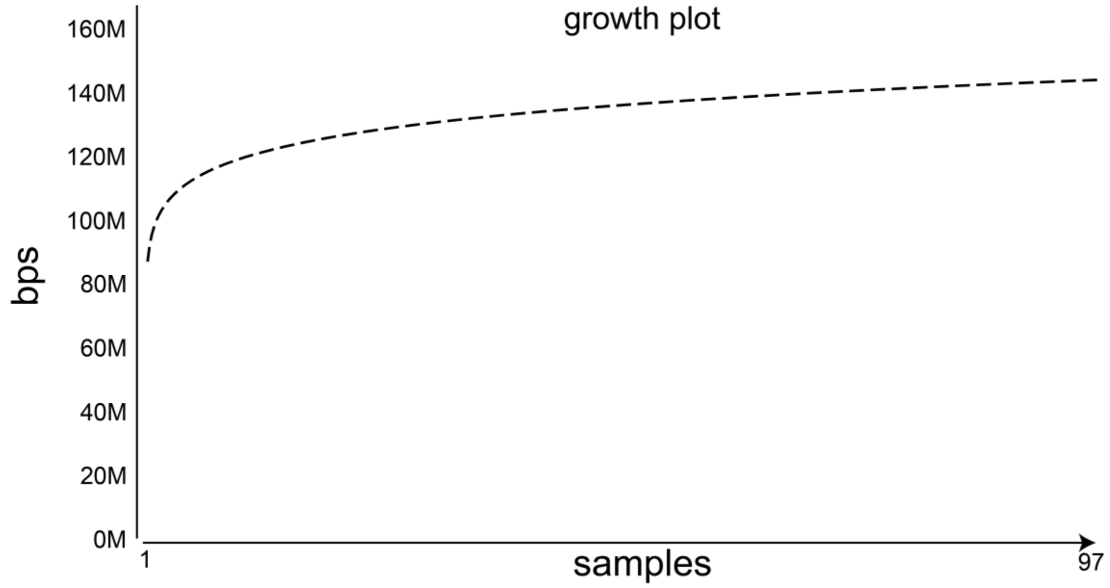

Supplemental Figure 4. Pangenome growth curves for 97 *A. thaliana*.

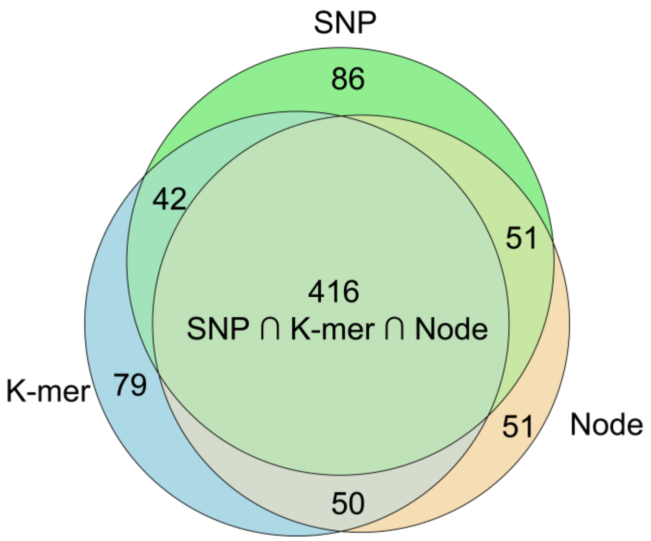

Supplemental Figure 5. Overlap between phenotypic traits and the hits derived from SNP, *k*-mer, and Node.

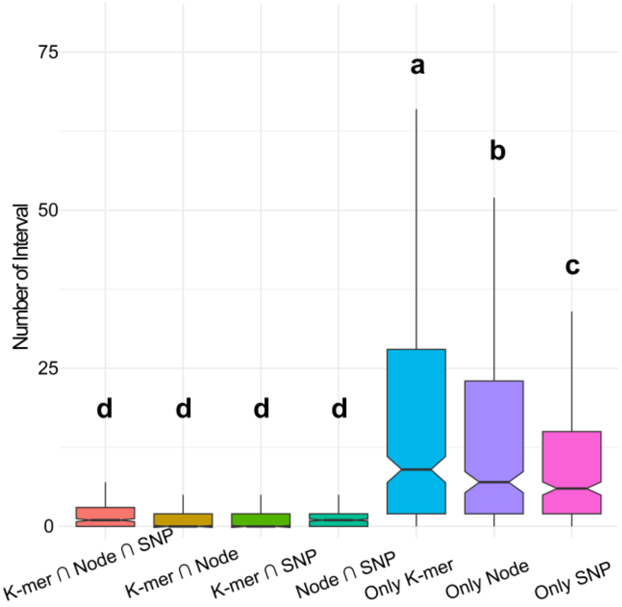

Supplemental Figure 6. Boxplot of the number of intervals in the intersection and union of three methods. Differences among groups were assessed using one-way ANOVA followed by LSD multiple comparison tests with Benjamini-Hochberg adjustment. Different letters indicate significant differences at $P < 0.05$.

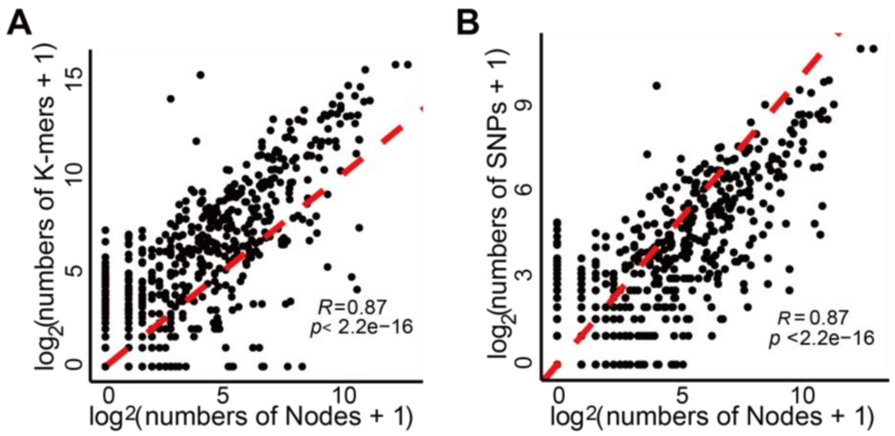

Supplemental Figure 7. Correlation between the number of significant markers. A: Correlation between the number of significant nodes and *k*-mers. B: Correlation between the number of significant nodes and SNPs. Pearson correlation coefficient (*R*) was calculated, and its significance (*P* values) was tested using t-tests.

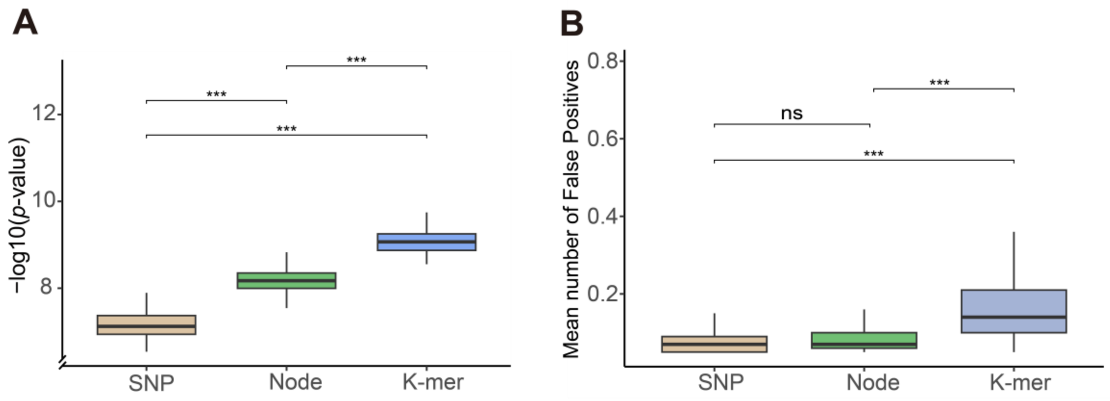

Supplemental Figure 8. Performance comparison of SNP, Node, and *k*-mer methods.

(A) Comparison of statistical significance. The y-axis represents -log10(*P*), with higher values indicating higher error rate. (B) Comparison of The Mean number of False Positives (MFP). The y-axis represents the MFP. Significance was calculated using the Wilcoxon test, "ns" indicates no significant difference, *** indicates $P \leq 0.001$.

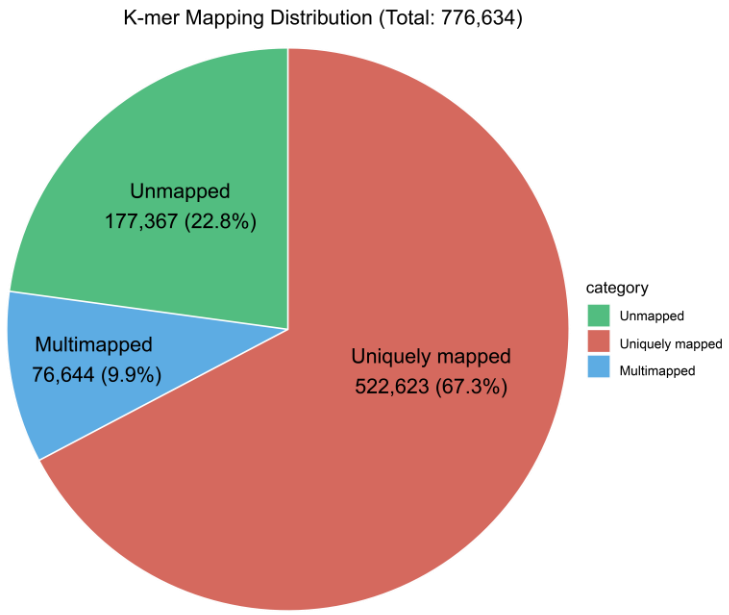

Supplemental Figure 9. The distribution of *k*-mer Mapping.

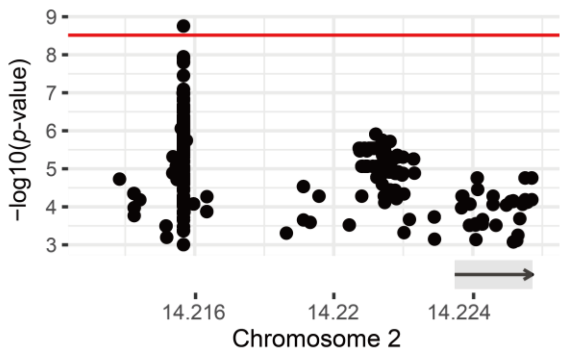

Supplemental Figure 10. Manhattan plot of a zoomed-in candidate region on chromosome 2, showing several significant loci located upstream of *AT2G27380*.

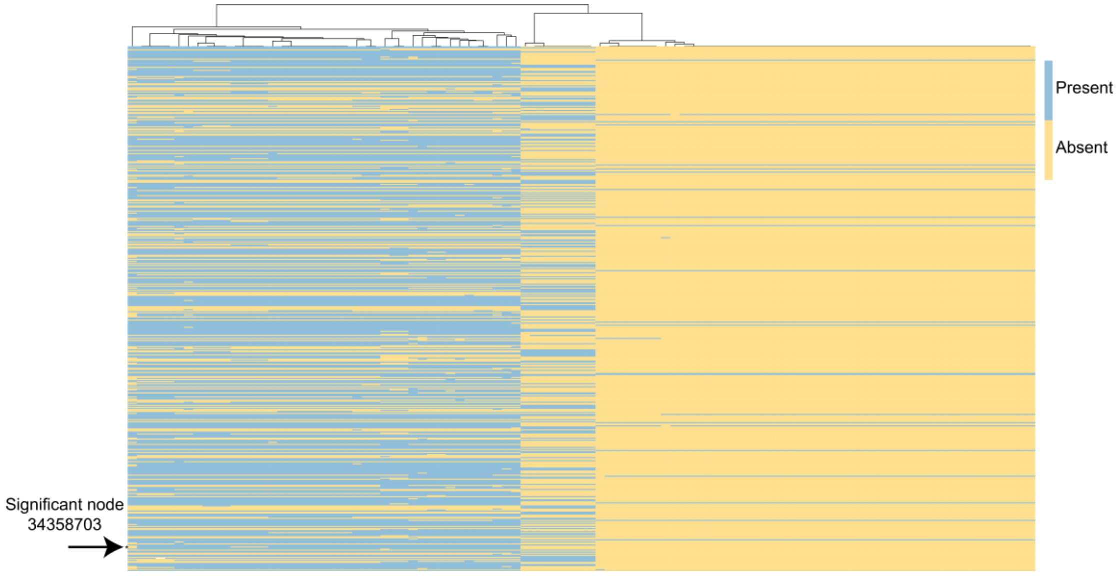

Supplemental Figure 11. Node-level presence/absence variation around the *EPR1* locus. Rows represent distinct nodes, while columns correspond to individual samples. The color scale denotes the node status: light blue represents presence, and light yellow represents absence. The arrow indicates a significant node identified by NodeGWAS.

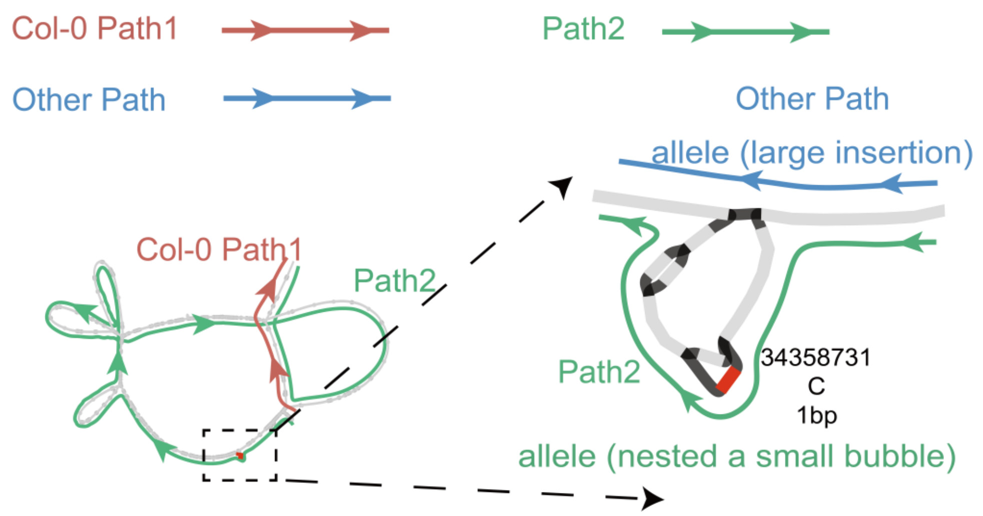

Supplemental Figure 12. Graph pangenome view of the associated locus (node ID: 34258731, sequence: C, length: 1 bp, highlighted in red). Left, overview of the local graph with alternative haplotype paths. Right, zoomed inset showing the red node as a single-base variant nested within a larger insertion (blue path). Arrows and colored paths indicate distinct haplotypes.

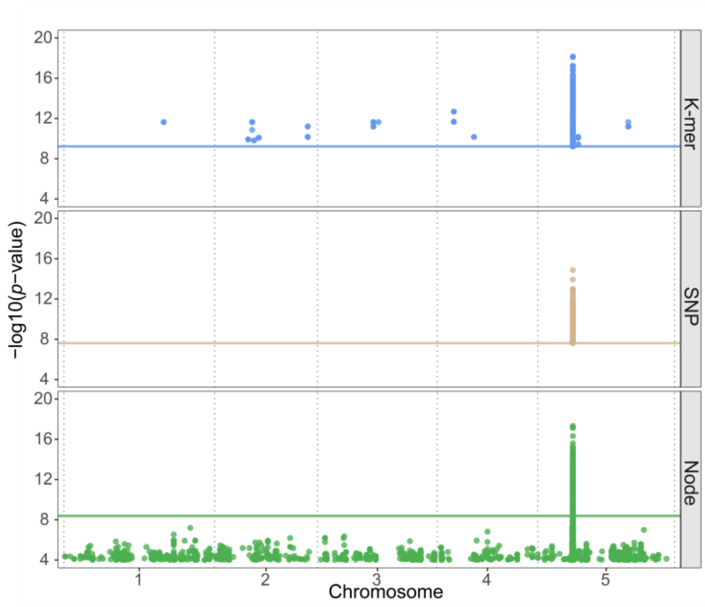

Supplemental Figure 13. Manhattan plots of quantitative disease resistance, with different colors representing different methods. Horizontal dashed lines indicate the genome-wide significance threshold used for each method. For the *k*-mer and SNP methods, only significant loci are displayed because the original *k*-merGWAS study only provided significant sites.

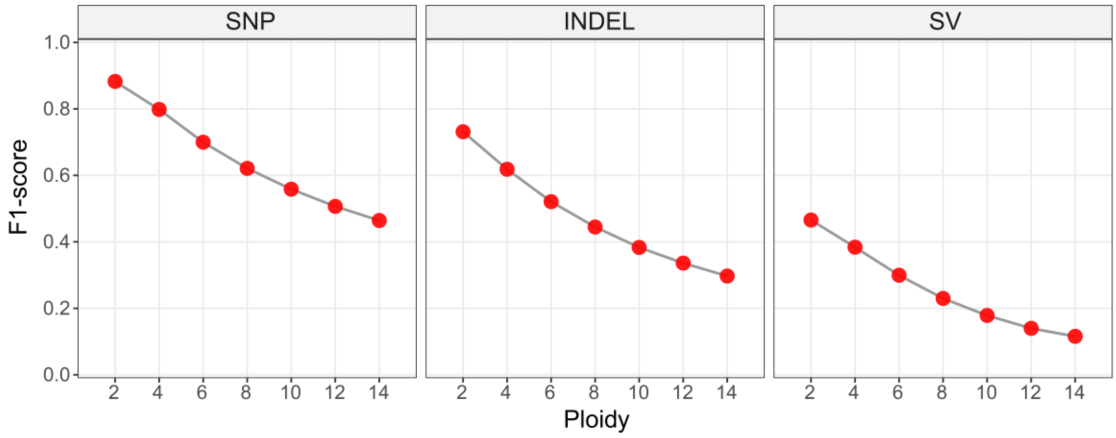

Supplemental Figure 14. F1-scores for variant detection across varying ploidy levels of *A. thaliana*, based on simulated sequencing data and a linear genome reference.

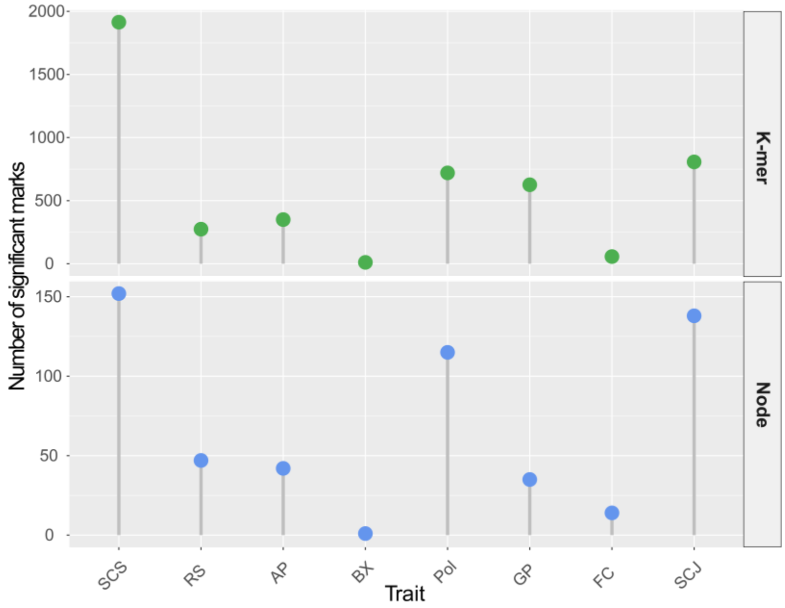

Supplemental Figure 15. Number of significant marks identified by SNP-, Node-, and *k*-mer-based methods. Sucrose-related traits. SCS: sucrose content in sugarcane; RS: reducing sugars; AP: apparent purity; BX: Brix; Pol: polarization; GP: gravity purity; FC: fiber content; SCJ: sucrose content in juice.

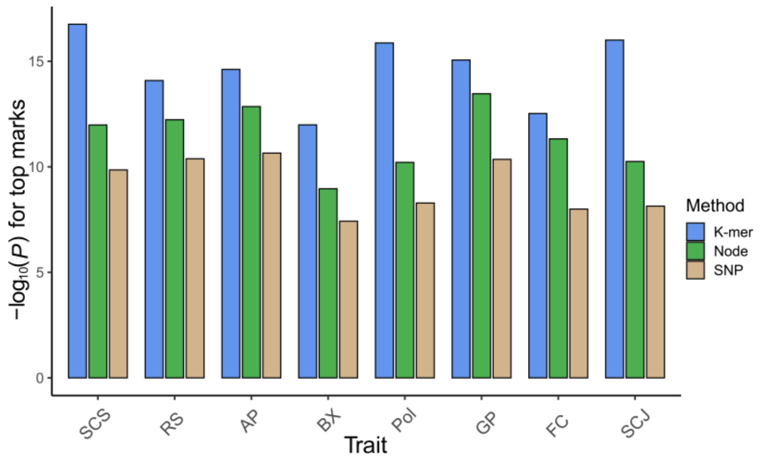

Supplemental Figure 16. *P* values of top marks identified by SNP-, Node-, and *k*-mer-based methods. Sucrose-related traits. SCS: sucrose content in sugarcane; RS: reducing sugars; AP: apparent purity; BX: Brix; Pol: polarization; GP: gravity purity; FC: fiber content; SCJ: sucrose content in juice.

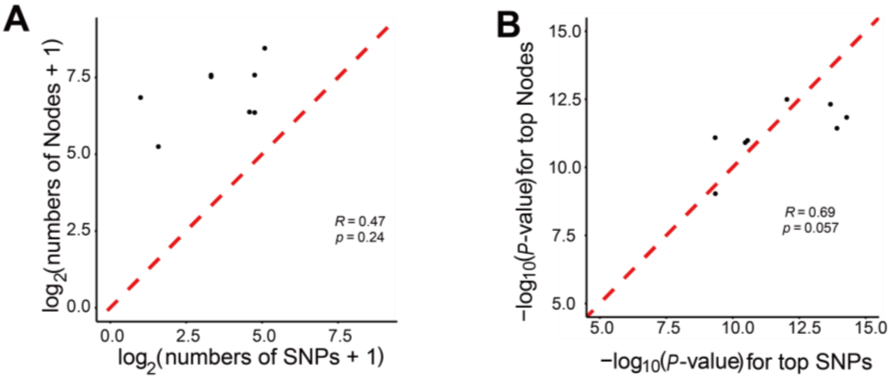

Supplemental Figure 17. Comparison of significant Nodes and significant SNPs.

A: Correlation between the number of significant Nodes and SNPs in sugarcane. B: Correlation of *P* values of top Nodes with SNPs in sugarcane. Pearson correlation coefficient (*R*) was calculated, and its significance (*P* value) was tested using t-tests.

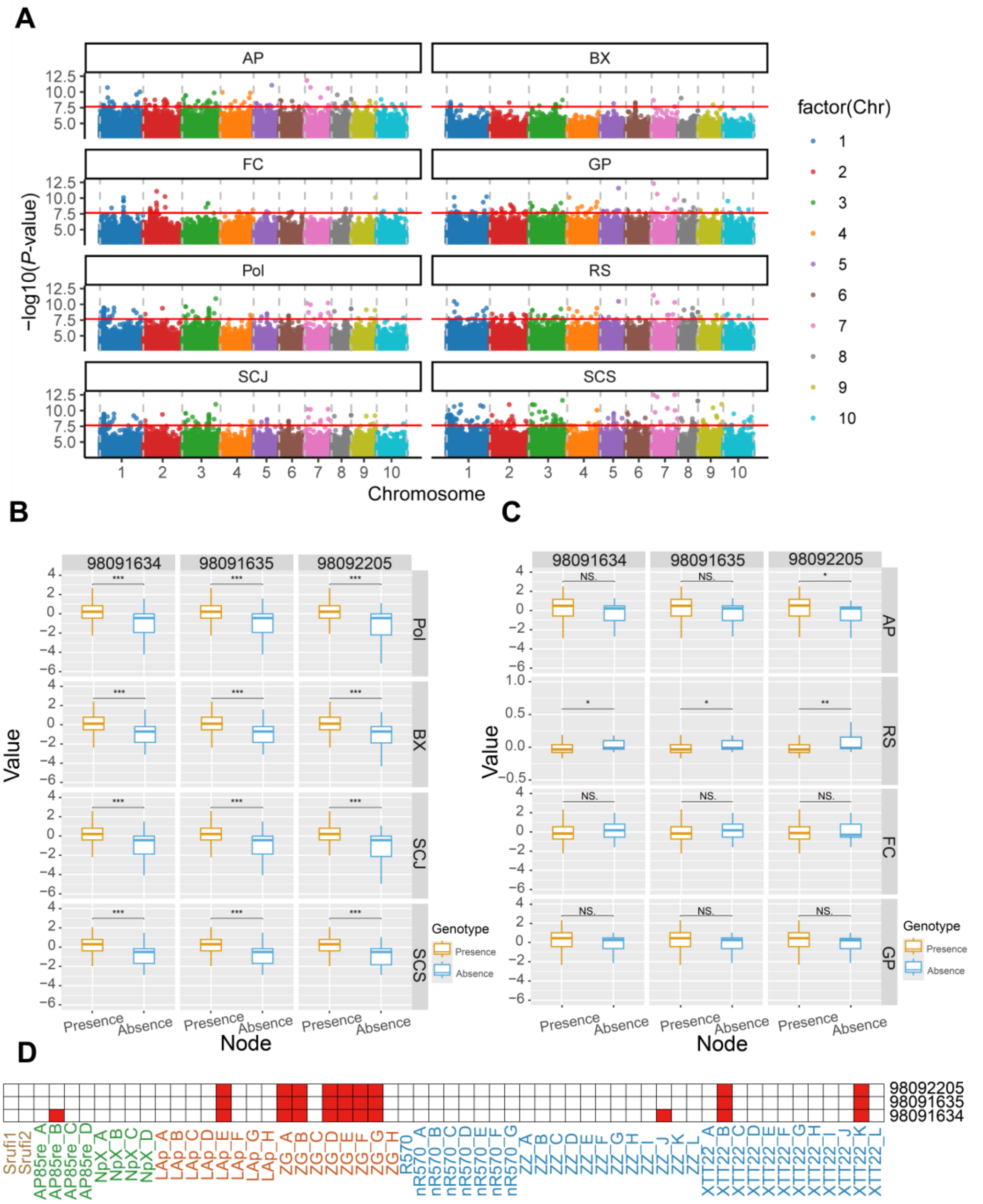

Supplemental Figure 18. GWAS identification of candidate loci for sucrose-related traits

A: Manhattan plot results for the NodeGWAS on 8 sugar-related traits. SCS: sucrose content in sugarcane; SCJ: sucrose content in juice; RS: reducing sugars; AP: apparent purity; GP: gravity purity; BX: Brix; Pol: polarization; FC: fiber content. **B-C:** Boxplot plot for 8 sucrose-related traits of three candidate nodes of *Erufi.06G007100*. significance (*P* value) was tested using t-tests, "NA" indicates no significant difference, *** indicates $P \leq 0.001$. **D:** The sources of three candidate nodes of *Erufi.06G007100*. The red block represents the node present in the haplotype.

Supplementary Table

Supplemental Table 1. Previous studies using *k*-merGWAS.

Species	Number of traits	Using *k*-mers Only	Reference
Arabidopsis, tomato, maize	2,000	YES	(Voichek and Weigel 2020)
wheat	2	NO	(Kale et al. 2022)
soybean	13	NO	(Lemay et al. 2023)
maize	4	YES	(He et al. 2024)
wheat	1	YES	(Zhang et al. 2024)
Lactuca sativa	1	YES	(Selvanayagam et al. 2025)

Supplemental Table 2. Definitions of terms in the graph pangenome used in this study.

Term	Definition
Node/Segment	A segment of DNA that is shared or unique among individuals. Depending on graph construction, nodes may be short (e.g., variant-scale) or represent longer haplotype segments.
Link/Edge	A connection linking two nodes (or segments) that are adjacent in one or more genomes. When the overlap is 0-bp, the B segment follows directly after A.
Path	A path is defined as an ordered sequence of nodes connected by edges. In a full pangenome graph, a path generally represents the complete chromosome of a specific individual. When restricted to a subgraph, the path represents a localized sequence segment. The biological interpretation of a path is an allele or haplotype.
Bubble (simple)	A bubble is a subgraph defined by two nodes, A and B, between which there exist at least two distinct paths. The different paths within the bubble represent alternative alleles at that locus. A bubble can encode simple variations, including SNPs, Indels, and SVs.
Nested bubble	Nested bubbles representing complex structural variations, where smaller variants are embedded within larger ones
Surject	Surject describes a computational projection of sequence alignments from a graph pangenome onto a linear reference genome, thereby translating graph-specific alignment formats (GAM/GAF) into standard linear alignment file formats (SAM/BAM).

Supplemental Table 3. Number of QTLs identified by *k*-merGWAS and NodeGWAS and their overlap.

Trait	*k*-mer	Node	Overlapped
SCS	173	38	9
RS	21	40	0
AP	36	1	1
BX	1	0	0
Pol	14	2	2
GP	41	30	1
FC	10	7	0
SCJ	56	26	2

Supplemental Table 4. Computing run time and memory usage of NodeGWAS and GATK.

GATK Pipeline	Real time (min)	CPU time (min)	Peak RAM (GB)	Threads
Read Mapping (BWA)	290.71	2,585.25	3.54	10
Coordinate Sorting	4.32	19.41	8.39	10
Mark Duplicates	20.67	28.86	30.99	10
Variant Calling	2,733.01	9,643.40	12.73	10
GenotypeGVCFs	65.41	65.24	1.19	10
Total time in one sample	3,114.12	12,342.16		
**NodeGWAS Pipeline**	**Real time (min)**	**CPU time (min)**	**Peak RAM (GB)**	**Threads**
Read Mapping (VG giraffe)	304.61	3,022.79	26.41	10
Coverage	67.7	185.57	26.79	10
Total time in one sample	372.31	3,208.36		
