# Supplementary material for: NodeGWAS: Leveraging graph pangenomes for sensitive and accurate association analyses across diverse diploid and polyploid species
Source: Plant Commun. 2026 Mar 25;7(7):101835. doi: 10.1016/j.xplc.2026.101835 (PMC13370226; doi:10.1016/j.xplc.2026.101835)
Supplement: Document S2. Article plus supplemental information [file mmc2.pdf]

Plant Communications
Correspondence

CellPress
Partner Journal

NodeGWAS: Leveraging graph pangenomes for sensitive and accurate association analyses across diverse diploid and polyploid species

Dear Editor,

This study introduces NodeGWAS, a graph pangenome-based framework that performs association analysis using graph nodes. Compared with conventional GWAS approaches, NodeGWAS strikes a balance between sensitivity and interpretability, and its applications in *Arabidopsis thaliana* and sugarcane demonstrate its effectiveness in dissecting complex traits.

Genome-wide association studies (GWAS) are essential for linking genomic variants to phenotypic traits (Tam et al., 2019). However, plant genomes vary widely in size, ploidy, heterozygosity, and repeat content (35%–90%), posing substantial challenges for genotyping (Wang et al., 2018). *k*-mer-based GWAS, which is reference-free, can detect a broader spectrum of variants beyond SNPs and insertions or deletions (Karikari et al., 2023) and has demonstrated strong performance compared with conventional approaches (Rahman et al., 2018; Voichek and Weigel, 2020) (Supplemental Table 1). However, it faces challenges in accurately mapping very short sequences to reference genomes and requires substantial computational resources (Supplemental Note 1).

In recent years, graph-based pangenomes have emerged as powerful alternatives to the standard linear reference genome, improving mapping accuracy and thereby enhancing downstream genotyping of both small variants and larger structural variants (Hickey et al., 2024). Nevertheless, studies on graph-based variant calling and genotyping remain limited (Supplemental Figure 1; Supplemental Table 2; Supplemental Note 2), whereas analytical approaches that directly leverage pangenome graph nodes hold considerable promise.

In this study, we propose NodeGWAS, a novel genotyping framework that operates directly on graph pangenomes and fills a critical gap in applying GWAS to species with high genetic diversity or complex structural variation—both of which pose substantial mappability challenges. In a graph pangenome, each node represents a non-redundant, variable-length "DNA word" (i.e., variable-length *k*-mers) that captures sequence variation across multiple genomes (Figure 1A; Supplemental Figures 2 and 3; Supplemental Table 2; Supplemental Note 3).

To comprehensively evaluate NodeGWAS, we applied it to a classic *Arabidopsis thaliana* population phenotype dataset, which includes 1,487 traits and 1,047 individuals (Rahman et al., 2018; Voichek and Weigel, 2020), and benchmarked it against both linear reference GWAS and *k*-mer-based approaches. Although the *A. thaliana* genome is relatively simple, it provides a suitable test case before extending NodeGWAS to more genetically complex species (Supplemental Figure 4; Supplemental Note 4).

A total of 775 traits with significant associations were identified across the three methods, of which 416 were consistently detected by all approaches, indicating substantial overlap (Supplemental Figure 5). Notably, NodeGWAS identified an additional 51 traits with significant associations that were not detected by the other methods. Using 16 randomly selected resequencing datasets, we further observed that, based on the graph pangenome, the alignment score increased from 96.98 to 99.00, and the mapping rate improved from 79.20% to 81.24%. The overlap of significant loci among the three methods was limited, and the number of regions uniquely identified by NodeGWAS fell between those identified by the *k*-mer- and SNP-based approaches (Supplemental Figure 6). In terms of sensitivity and statistical significance, the number of significant variants and the top *pp* values were highly correlated across methods (R = 0.67–0.87; Figures 1B and 1C; Supplemental Figures 7A and 7B). NodeGWAS outperformed the SNP-based method but showed lower sensitivity than the *k*-mer-based approach (Supplemental Note 5). Although *k*-mer-based methods exhibit higher apparent sensitivity to GWAS signals, our results indicate that NodeGWAS provides superior control of false positives compared with unstructured *k*-mer approaches (Supplemental Figures 8A and 8B; Supplemental Note 6).

It is worth noting that, due to the inherent properties of *k*-mers, a single variant may generate dozens or even hundreds of *k*-mers, leading to substantial redundancy and potential over-reporting. In contrast, NodeGWAS reports only polymorphic nodes that are in linkage disequilibrium. *k*-merGWAS identified 776,634 significant *k*-mers across 1,487 traits, of which 33.7% could not be reliably assigned to the reference genome (Supplemental Figure 9). By contrast, NodeGWAS leverages graph structure and node context (e.g., the link field in graphical fragment assembly (GFA); Supplemental Figure 2; Supplemental Table 2) to map all 87,510 significant nodes to reference coordinates, whereas the conventional SNP-based method identified only 28,954 loci. Overall, NodeGWAS achieves a balance between sensitivity and interpretability.

We further evaluated NodeGWAS using the canonical flowering time trait (10°C) in *A. thaliana* and successfully recapitulated all well-established loci, including *Flowering Locus T*, *Short Vegetative Phase*, *Flowering Locus C*, *Delay of Germination 1*, and *Vernalization Insensitive 3* (Figure 1D). Additionally, NodeGWAS identified two signal intervals that were not detected by either the linear reference or *k*-mer-based approaches. Near the *VIN3* locus, NodeGWAS identified 55 significant nodes. In comparison,

Plant Communications 7, 101835, July 13 2026 © 2026 The Author(s). Published by Elsevier Inc. on behalf of CAS Center for Excellence in Molecular Plant Sciences, Chinese Academy of Sciences, and Chinese Society for Plant Biology.
This is an open access article under the CC BY license (http://creativecommons.org/licenses/by/4.0/).

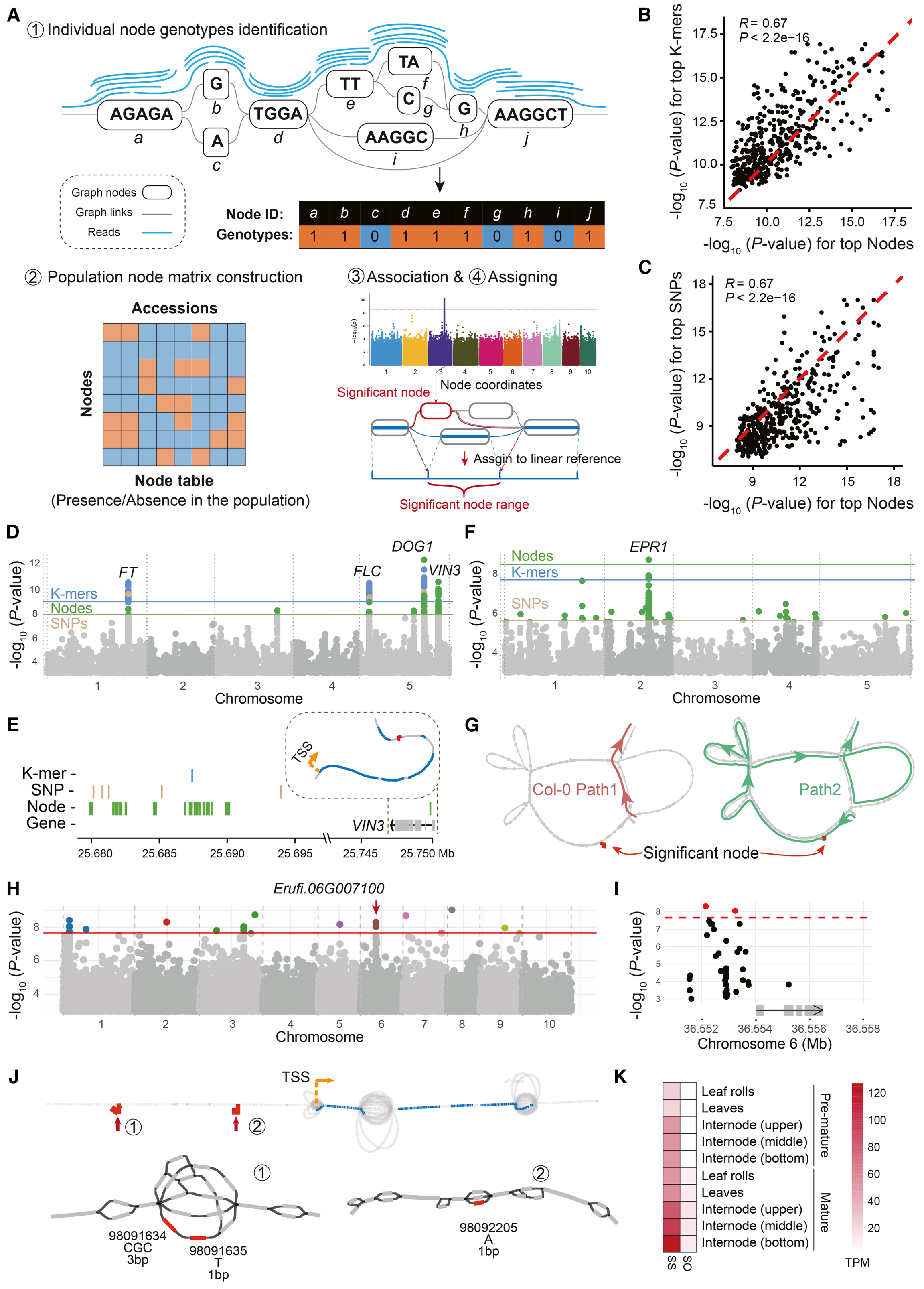

Figure 1. NodeGWAS: Workflow, performance evaluation, and application.

(A) NodeGWAS workflow. **(1)** Individual node genotyping: Resequencing reads are aligned to the pangenome graph to determine the presence (1, orange) or absence (0, blue) of nodes (e.g., node *a* = "AGAGA"). **(2)** Matrix construction: Node genotypes from all individuals are compiled into a population-level

(legend continued on next page)

k-merGWAS detected only two significant *k*-mers, with just one mapping to chromosome 5 and the other remaining unmapped. SNP-based GWAS identified five significantly associated SNPs in the downstream regulatory region of *VIN3*. Notably, one of the significant nodes was located within an intron of *VIN3*, suggesting a novel causal variant that may influence gene function (Figure 1E).

NodeGWAS revealed distinct advantages for detecting certain traits. For instance, we identified novel significant loci near *Early Phytochrome Response 1* associated with root length responses to iron availability, which were not detected by *k*-mer- or SNP-based GWAS (Figure 1F; Supplemental Figure 10; Satbhai et al., 2017; Kumari et al., 2019). Iron may regulate phytochrome function by influencing the cellular redox state or modulating iron–sulfur clusters, thereby affecting root growth (Rockwell and Lagarias, 2020). By examining the positions of significant nodes, we identified 30 distinct paths among the 97 samples (Supplemental Figures 2 and 11; Supplemental Table 2; Supplemental Note 7). Notably, eight samples followed path 2, which contains a large insertion relative to path 1 (Figure 1G). The presence of the significant node within path 2 is associated with increased root length. This node is located within a small bubble inside the inserted fragment (highlighted in red in Supplemental Figure 12), which may complicate the detection of large structural variants and lead to failure to detect these loci. This observation provides empirical support for the 'nested variation' concept described above. Additionally, NodeGWAS demonstrated improved control of false positives for certain traits (Supplemental Figure 13; Supplemental Note 8).

Compared with the small, relatively simple diploid genome of *A. thaliana*, genotyping complex polyploids has long been plagued by high error rates and difficulties in comparing individuals across varying ploidy levels, severely limiting its downstream applications in population genetics (Supplemental Figure 14; Phillips, 2024). By introducing a graph-based pangenome reference, NodeGWAS mitigates the alignment bias associated with a single reference, captures comprehensive genetic diversity, and effectively resolves alignment challenges in polyploids. It also sidesteps common errors inherent to traditional polyploid genotyping while maintaining the accuracy and completeness of genetic information by using node coverage/counts as GWAS predictors. Consequently, NodeGWAS is naturally adaptable to species with varying ploidy levels.

To illustrate this advantage, we applied NodeGWAS to sugarcane, a modern cultivated crop with a highly polyploid genome. This genome is derived from interspecific hybridization among multiple *Saccharum* species or varieties and is among the most complex and diverse crop genomes (Zhang et al., 2025). Owing to its variable ploidy levels and hybrid composition, accurate genotyping in sugarcane remains a considerable challenge. We performed permutation tests to ensure rigorous false-positive control and fair comparisons (Supplemental methods). Across eight sugar-related traits, SNP-based GWAS yielded no significant associations, whereas *k*-mer-based and NodeGWAS approaches identified 4,751 and 544 loci, respectively (Supplemental Figure 15). Statistical power followed the order *k*-mer > node > SNP (Supplemental Figure 16). The limited overlap between *k*-mer and NodeGWAS results (e.g., only nine shared quantitative trait loci for sucrose content) indicates that NodeGWAS provides a complementary approach by capturing distinct genomic signals (Supplemental Table 3). Compared with previous allele-level linear reference studies (Zhang et al., 2025), NodeGWAS identified more significant loci and yielded stronger statistical signals (Supplemental Figure 17). In total, NodeGWAS detected 143 loci across more than half of the traits. For example, at ∼36 Mb on chromosome 6, we identified three nodes significantly associated with four of the eight traits (Figure 1H; Supplemental Figure 18A). The presence of these nodes significantly increases the four sugar-related traits (Supplemental Figures 18B and 18C), and they are predominantly found in *Saccharum officinarum* (Supplemental Figure 18D), which contributes high sugar content to modern cultivars. Using a pangenome subgraph and coordinate conversion to a linear reference genome, we determined that these three loci reside in the promoter region of *Erufi.06G007100* (Figures 1I and 1J), which encodes a glucuronosyltransferase and is highly homologous to *IRREGULAR XYLEM 10*. Transcriptome analysis further showed that *Erufi.06G007100* is consistently expressed at lower levels in

matrix. **(3)** Association analysis: GWAS identifies nodes significantly associated with the trait. **(4)** Node assignment: Significant nodes are projected onto the linear reference genome to facilitate candidate gene discovery.

(B and C) Correlation of top *p* values between top nodes and *k*-mers **(B)** or SNPs **(C)**. Pearson correlation coefficients (*R*) and corresponding *p* values were calculated using *t*-tests.

(D) Manhattan plots of the flowering-time trait using three different methods. Only significant *k*-mers and SNPs are shown. *FLOWERING LOCUS T* (*FT*), *FLOWERING LOCUS C (FLC), DELAY OF GERMINATION 1* (*DOG1*), *and VERNALIZATION INSENSITIVE 3* (*VIN3*) are genes from *A. thaliana*.

(E) Genomic positions of significant markers from three GWAS approaches. A graph pangenome representation of the *AT5G57380* locus and its flanking regions is shown. NodeGWAS identifies a significant node (red dot) within an intronic region. The orange arrow indicates the transcription start site (TSS), and coding sequences are shown in blue.

(F) Manhattan plots of the root-length trait analyzed using three methods. Only significant *k*-mers and SNPs are shown. *EARLY PHYTOCHROME RESPONSE 1 (EPR1)* is a gene from *A. thaliana*.

(G) Allelic paths near *EPR1* in the wild-type Col-0 and an alternative allele path (path 2), which contains the significant node (Node 34358731; red dot).

(H) Manhattan plots of sucrose-content traits in sugarcane generated by NodeGWAS. A red arrow indicates significant signals near the *Erufi.06G007100* locus.

(I) Zoomed-in Manhattan plot of a candidate region on chromosome 6, showing multiple significant loci in the TSS region of *Erufi.06G007100*.

(J) Graph pangenome representation of the *Erufi.06G007100* locus and its flanking regions. Top: subgraph visualization with significant nodes indicated by red arrows. The orange arrow marks the TSS, and coding sequences are shown in blue. Bottom: enlarged view of significant nodes (red) and their flanking nodes. Significant nodes are labeled by node ID/sequence/length (e.g., 98091634/CGC/3 bp, 98091635/T/1 bp, and 98092205/A/1 bp).

(K) Expression heatmap of *Erufi.06G007100* in *Saccharum spontaneum* (Ss) and *Saccharum officinarum* (So). Gene expression levels are shown as TPM (transcripts per million), normalized for gene length and sequencing depth.

stems and leaves of *S. officinarum* across developmental stages compared with *Saccharum spontaneum* (Figure 1K), implicating a regulatory role in sugar accumulation.

In this study, we present NodeGWAS, a novel strategy for performing GWAS directly on graph-based genotypes defined by structurally resolved nodes. Using this end-to-end workflow, we demonstrate that NodeGWAS reduces redundancy and false positives and uncovers novel candidate genes in *A. thaliana* that are missed by traditional approaches. We further extend NodeGWAS to polyploid sugarcane, where it identifies highly significant functional loci associated with sugar-content traits that would otherwise remain undetected. Overall, our work provides a robust and interpretable framework applicable to both diploid and polyploid genomes (Supplemental Note 9).

DATA AND CODE AVAILABILITY

The code and full documentation are available on GitHub: https://github.com/zhangyixing3/NodeGWAS.

FUNDING

This work was supported by the National Key Research and Development Program (2024YFF1000800 to H.T. and J.Z.) and the National Natural Science Foundation of China (grant 32500548 to Y.H.).

ACKNOWLEDGMENTS

No conflict of interest is declared.

AUTHOR CONTRIBUTIONS

H.T., Y.H., and J.Z. conceived and designed the project. Y.Z. performed the main analyses, drafted the initial manuscript, and implemented the pipeline in Rust. Yuheng Wang, T.W., Y.L., Y.T., Y.Q., Yuhao Wang, B. W., and Z.W. contributed to data analysis and pipeline evaluation. H.T., Y.H., J.Z., and Q.Z. revised the manuscript. All authors read and approved the final version.

SUPPLEMENTAL INFORMATION

Supplemental information is available at *Plant Communications Online*.

Received: May 21, 2025
Revised: June 25, 2025
Accepted: March 23, 2026
Published: March 25, 2026

Yixing Zhang[1], Yuheng Wang[1], Tingting Wu[1], Yan Lin[1], Yue Tan[1], Yiying Qi[1], Yuhao Wang[2], Baiyu Wang[2], Zhengguang Wang[1], Qing Zhang[2], Jisen Zhang[2,], Yumin Huang[1,*] and Haibao Tang[1,*]*

[1]Fujian Provincial Key Laboratory of Haixia Plant Systems Biology, Haixia Institute of Science and Technology & College of Life Science, Fujian Agriculture and Forestry University, Fuzhou 350002, China
[2]State Key Lab for Conservation and Utilization of Subtropical Agro-Biological Resources, Guangxi Key Lab for Sugarcane Biology, College of Agriculture, Guangxi University, Nanning 530004, China
***Correspondence: Jisen Zhang (zjisen@126.com), Yumin Huang (ymhuang@fafu.edu.cn), Haibao Tang (tanghaibao@gmail.com)**
https://doi.org/10.1016/j.xplc.2026.101835

REFERENCES

Hickey, G., Monlong, J., Ebler, J., Novak, A.M., Eizenga, J.M., Gao, Y., Human Pangenome Reference Consortium, Marschall, T., Li, H., and Paten, B. (2024). Pangenome Graph Construction from Genome Alignments with Minigraph-Cactus. Nat. Biotechnol. **42**:663–673.

Karikari, B., Lemay, M.-A., and Belzile, F. (2023). K-Mer-Based Genome-Wide Association Studies in Plants: Advances, Challenges, and Perspectives. Genes **14**:1439.

Kumari, S., Yadav, S., Patra, D., Singh, S., Sarkar, A.K., and Panigrahi, K.C.S. (2019). Uncovering the Molecular Signature Underlying the Light Intensity-Dependent Root Development in Arabidopsis Thaliana. BMC Genom. **20**:596.

Phillips, A.R. (2024). Variant Calling in Polyploids for Population and Quantitative Genetics. Appl. Plant Sci. **12**:e11607.

Rahman, A., Hallgrímsdóttir, I., Eisen, M., and Pachter, L. (2018). Association Mapping from Sequencing Reads Using K-Mers. eLife **7**: e32920. https://doi.org/10.7554/eLife.32920.

Rockwell, N.C., and Lagarias, J.C. (2020). Phytochrome Evolution in 3D: Deletion, Duplication, and Diversification. New Phytol. **225**:2283–2300.

Satbhai, S.B., Setzer, C., Freynschlag, F., Slovak, R., Kerdaffrec, E., and Busch, W. (2017). Natural Allelic Variation of FRO2 Modulates Arabidopsis Root Growth under Iron Deficiency. Nat. Commun. **8**:15603.

Tam, V., Patel, N., Turcotte, M., Bossé, Y., Paré, G., and Meyre, D. (2019). Benefits and Limitations of Genome-Wide Association Studies. Nat. Rev. Genet. **20**:467–484.

Voichek, Y., and Weigel, D. (2020). Identifying Genetic Variants Underlying Phenotypic Variation in Plants without Complete Genomes. Nat. Genet. **52**:534–540.

Wang, P., Moore, B.M., Panchy, N.L., Meng, F., Lehti-Shiu, M.D., and Shiu, S.-H. (2018). Factors Influencing Gene Family Size Variation among Related Species in a Plant Family, Solanaceae. Genome Biol. Evol. **10**:2596–2613.

Zhang, J., Qi, Y., Hua, X., Wang, Y., Wang, B., Qi, Y., Huang, Y., Yu, Z., Gao, R., Zhang, Y., et al. (2025). The Highly Allo-Autopolyploid Modern Sugarcane Genome and Very Recent Allopolyploidization in Saccharum. Nat. Genet. **57**:242–253.

Plant Communications, Volume 7

Supplemental information

NodeGWAS: Leveraging graph pangenomes for sensitive and accurate association analyses across diverse diploid and polyploid species

Yixing Zhang, Yuheng Wang, Tingting Wu, Yan Lin, Yue Tan, Yiying Qi, Yuhao Wang, Baiyu Wang, Zhengguang Wang, Qing Zhang, Jisen Zhang, Yumin Huang, and Haibao Tang

NodeGWAS: Leveraging graph pangenomes for sensitive and accurate association analysis across diverse diploid and polyploid species

Yixing Zhang[1], Yuheng Wang[1], Tingting Wu[1], Yan Lin[1], Yue Tan[1], Yiying Qi[1], Yuhao Wang[2], Baiyu Wang[2], Zhengguang Wang[1], Qing Zhang[2], Jisen Zhang[2,*], Yumin Huang[1,*], Haibao Tang[1,*]

[1.] Fujian Provincial Key Laboratory of Haixia Plant Systems Biology, Haixia Institute of Science and Technology & College of Life Science, Fujian Agriculture and Forestry University, Fuzhou, 350002 China.
[2.] State Key Lab for Conservation and Utilization of Subtropical Agro-Biological Resources, Guangxi Key Lab for Sugarcane Biology, College of Agriculture, Guangxi University, Nanning 530004, China.

* To whom correspondence may be addressed. Email: zjisen@126.com, ymhuang@fafu.edu.cn or tanghaibao@gmail.com

Supplemental Methods

Pangenome construction

69 *A. thaliana* accessions were downloaded from NCBI (NCBI BioProject accession: PRJNA1033522) (Lian et al. 2024). 32 *A. thaliana* accessions were downloaded from Figshare (https://figshare.com/articles/dataset/32_ecotypes_Arabidopsis_thaliana_genomes_gene_annotation_pan-TE_library_graph_pan-genome_gene_family_and_gene_presence_absence_matrices_files_/21673895) (Kang et al. 2023). The T2T *A. thaliana* genome was downloaded from GitHub (https://github.com/schatzlab/Col-CEN) (Naish et al. 2021).

The graph-based pangenome of *A. thaliana* was constructed using 97 non-redundant genome assemblies through the Minigraph-Cactus pipeline (version 5.12.0) under parameters of "--reference 035_Col_CEN_v12 --filter 10 --vcf full clip filter --giraffe --gfa full clip filter --gbz full clip filter --draw full clip --viz full clip --odgi full clip filter", with the telomere-to-telomere (T2T) *A. thaliana* genome serving as the reference. To align the resequencing data, the GFA graph, which was filtered by removing nodes covered by fewer than 10 haplotypes, was used.

Using the PGGB pipeline (version 0.5.2), we constructed a pangenome graph incorporating the AP85-441, LA-purple, Np-X, ZG, XTT22, R570 and *Erianthus rufipilus* genomes, which was subsequently decomposed into VCF files (Zhang et al. 2025; Q. Zhang et al. 2022; Zhang et al. 2018; Wang et al. 2023; Healey et al. 2024). These VCF files were then merged with VCF data from 60 *E. rufipilus* accessions, and a comprehensive pangenome was constructed using the *vg autoindex* tool with default parameters based on the linear reference genome of *E. rufipilus* (Garrison et al. 2018; Wang et al. 2023; Huang et al. 2026).

Data alignment

Resequencing data for 1,047 *A. thaliana* accessions were obtained from the NCBI Sequence Read Archive (SRA accession: SRP056687) (1001 Genomes Consortium. Electronic address: magnus.nordborg@gmi.oeaw.ac.at and 1001 Genomes Consortium 2016). For sugarcane, whole-genome resequencing data for 183 accessions were downloaded from the SRA (Accessions: SRR29827487-SRR29827669).

The resequencing reads were aligned to the pangenome graph using *vg giraffe* (version v1.61.0) with default settings (Sirén et al. 2021). The mapping results were filtered using *vg filter* with the parameters: -r 0.90 -fu -m 1 -q 15 -D 999. The filtration criteria were specifically devised to retain high-quality alignments with mapping quality exceeding 15, a primary alignment score of at least 0.90, and at least one match at each terminus, enabling the trimming of reads.

Computational resources and benchmarking

To demonstrate the practical efficiency of our framework, we directly compared the computational demands of NodeGWAS and GATK (using the "-ploidy 4" mode) on a real tetraploid potato population dataset (F. Zhang et al. 2022). All analyses were performed on a computing node equipped with 128 CPU cores and 512 GB RAM. The results show that GATK required approximately 51.9 hours (3,114 minutes) to process a single sample, whereas NodeGWAS completed the same task in only 6.2 hours (372 minutes), an approximately 8.36-fold faster performance (Supplemental Table 4). Peak memory usage of NodeGWAS (26.79 GB) was comparable to GATK (30.99 GB) in polyploid mode and remained within highly accessible ranges (Supplemental Table 4).

Permutation testing for k-mers, SNPs, and nodes in A. thaliana

Like *k*-mer-based GWAS, the EMMA (emma.REMLE function, emma.R) was employed to estimate the variance components, which were subsequently used to compute the phenotypic covariance matrix (Kang et al. 2008). We then performed 100 permutations of the phenotype using the mvnpermute R package (version 1.0.1) (Abney 2015). The family-wise error rate (FWER) threshold at *n*% (for instance, *n*=5 corresponds to a 5% threshold) was determined by selecting the *n*-th top *P* value from the 100 top *P* values obtained from performing genome-wide association analysis on each permutation.

Genome-wide association analysis of A. thaliana

In NodeGWAS, each alignment file was converted to JSON format by *vg view*, which enabled us to directly process the alignment information for each read and count the occurrences of nodes that are perfectly matched by the reads. Nodes with a count of two or more were considered as "presence", while counts fewer than two were considered as "absence". Finally, we combined the presence/absence information for all nodes from all accessions and generated a presence/absence matrix. The status of presence/absence of nodes was converted to biallelic genotypes for association. Nodes with minor allele frequency (MAF) < 0.05 were filtered. GWAS was performed by LMMs with the kinship matrix in GEMMA (version 0.98.5) (Zhou and Stephens 2012).

A. thaliana GWAS results including *k*-mer and SNP analyses were downloaded from Zenodo (https://zenodo.org/records/3701176#.XmX9u5NKhhE) (Voichek and Weigel 2020).

Genome-wide association analysis of Saccharum spp.

Like *A*. *thaliana*, we obtained the filtered genotype files. GWAS was performed by Mixed-Model Association eXpedited program (EMMAX, version 20120210) (Kang et al. 2010), with the first ten principal components (PCs) included as a random effect matrix. The genome-wide significance threshold was determined by Bonferroni correction and was set to 2.19068e-08. We also evaluated the performance between NodeGWAS and SNP, *k*-mer-based GWAS. To ensure rigorous false-positive control and establish fair significance thresholds, we performed 100 permutation tests by randomly shuffling phenotypes while

keeping genotype and kinship matrices constant. The 5th percentile of the minimum *P* values from permutations defines the significant threshold.

Conversion of genomic coordinates

For each trait, we extracted the genomic regions within ±5000 bp of the significantly associated loci using bedtools (version 2.30.0) and merged overlapping regions with bedtools merge to obtain non-redundant genomic intervals (Quinlan and Hall 2010). To further compare the distribution patterns of associated loci identified by the *k*-merGWAS, NodeGWAS and SNP-based methods, we employed bedtools intersect to quantify the overlapping regions among different methods and bedtools subtract to identify method-specific genomic regions.

Inject back into the linear genome

For nodes located on the linear reference genome (e.g., the T2T Col *Arabidopsis* reference genome), we determine their coordinates by calculating the cumulative length offset of each node along the reference sequence. For nodes that are not part of the linear reference genome (such as those formed by insertions or other variants), we locate the genomic position at which the variant represented by the node is inserted/deleted into the reference genome and use that position as the node’s coordinate.

Supplementary Notes

1. Limitations of k-mer-based GWAS

Accurately linking extremely short fixed-length *k*-mer sequences (e.g., 31 bp) to specific genomic regions in plant reference genomes remains a significant challenge, particularly in species with high ploidy levels or abundant repetitive sequences. For example, using *k*-merGWAS, only 1,542 (5.4%) and 3,929 (3.6%) significant *k*-mers could be directly and accurately mapped to the reference genome, for maize kernel oil content and leaf angle trait, respectively (He et al. 2024). On the other hand, compared to traditional genetic markers, *k*-mer analysis requires substantially more computational resources (Karikari et al. 2023). An example is the detection of 1.1 billion *k*-mers from 261 inbred lines, with the majority of lines containing 0.35–0.55 billion *k*-mers (He et al. 2024). Such a large volume and complexity of data presents considerable challenges for computation.

2. Challenges in graph-based genotyping

Traditional graph-based genotyping methods (such as PanGenie, *vg call*, etc.) (Hickey et al. 2020; Ebler et al. 2022) are primarily optimized for diploid genomes, which are fundamentally incompatible with the complex allele dosage effects in polyploids. Node-based analysis "sidesteps" this by using graph node coverage/counts as predictors (Supplemental Figure 1). This makes it naturally adaptable to species with varying ploidy levels. Meanwhile, large structural variants (SVs) are particularly challenging, as they often form complex, nested ‘bubble’ structures (Supplemental Table 2) that are both difficult to detect and also poorly represented in the VCF format (Romain et al. 2025). Node-based analysis avoids this by decomposing complex topologies into their most basic units—nodes. It does not need to decide whether a complex region is “one SV” versus “multiple SVs,” nor does it require stable breakpoints in a VCF representation (Supplemental Figure 1). Finally, traditional graph-based variant calling typically surjects read alignments (Supplemental Table 2) onto a chosen linear reference genome and then is used with conventional linear-based variant callers. Node-based analysis bypasses the severe information loss inherent to traditional linear-based SV calling pipelines. Furthermore, this two-stage workflow is cumbersome, as it requires handling SNPs and SVs through separate pipelines.

3. NodeGWAS workflow

In a sequence-resolved variation graph, any sequence-level variation (including SNPs, INDELs and complex SVs) triggers the creation of distinct, alternative nodes, and is encoded within the topology of the graph. NodeGWAS treats each node as an independent genetic unit. The 0/1 genotype matrix successfully distinguishes these variants and captures their distinct biological implications (Supplemental Figure 3). Thus, our node-based association analysis preserves the full spectrum of genomic variation. This "node-as-a-feature" approach allows NodeGWAS to perform association mapping across the entire genomic spectrum without the need for explicit SV calling, thereby bypassing reference bias and complexity constraints. NodeGWAS follows a four-stage workflow

(Figure 1A): (1) Align each individual resequencing data to the graph pangenome to identify the genotypes (presence or absence) of each node, (2) Collate the node genotypes of all individuals to build a population-level node matrix (3) Perform GWAS analysis using node matrix, with appropriate covariate and population-structure controls, to identify significantly associated nodes. (4) Assign node coordinates back to the linear reference genome for downstream annotation. NodeGWAS is agnostic of the graph pangenome construction methods and can directly work with prebuilt pan-genomes constructed with Minigraph-Cactus, PGGB, or Vg (Hickey et al. 2024; Garrison et al. 2024, 2018).

4. A. thaliana graph pangenome

We first constructed a high-quality *A. thaliana* graph pangenome using the Minigraph-Cactus pipeline, which incorporates 97 diverse assemblies from previous studies (Lian et al. 2024; Kang et al. 2023). The growth curve, generated using Panacus to model the relationship between the number of genomes included and the total graph size, indicated that the pangenome has reached saturation at 97 genomes (Supplemental Figure 4) (Parmigiani et al. 2024). This suggests that the graph-based pangenome has effectively captured the genetic diversity of the Arabidopsis population and can serve as a comprehensive reference for downstream analyses.

5. Comparison of GWAS methods in significant loci detection

Compared to the SNP-based approach, NodeGWAS identified more significant loci in 27.3% of the traits, whereas the SNP-based method did so in only 16.6%. In comparison to the *k*-mer-based approach, NodeGWAS detected fewer significant loci in only 32.9% of the traits (*k*-mer-based: 12.4%). In terms of association strength, NodeGWAS exceeded the top-hit signals of the SNP-based method in 29.4% of the traits, with the remaining (15.8%) showing lower significance. Similarly, compared to the *k*-mer-based method, NodeGWAS demonstrated stronger signals in 15.2% of the traits (*k*-mer-based: 29.4%).

6. False Positive Assessment via Permutation Tests

To evaluate the accuracy across different methods in *A. thaliana*, we randomly selected 125 traits and performed 100 phenotype permutations for each, while maintaining the original genotype matrix and covariates.

The Mean number of False Positives (MFP) is defined as the total number of significant loci observed across 100 permutation tests divided by 100:

$$\mathrm{FPR} = \frac{\sum_{i=1}^{100} N_i}{100}$$

where N_i is the number of significant loci detected in the i-th permutation.

we observed that the *k*-mer-based method suffers from the highest error rate, followed by Node, with SNP the lowest (Supplemental Figure 8A, Wilcoxon test, *P* < 0.001). The SNP- and Node-based methods have similarly low Mean number of False Positives (MFP) with

no significant difference, whereas the *k*-mer-based method exhibits a significantly higher MFP than the others (Supplemental Figure 8B, Wilcoxon test, $P < 0.001$).

7. paths near the significant nodes

The total length ranges from 125 to 1,956 bp. These paths are not structurally independent, as multiple paths often share common subsequences, which are represented as shared nodes in the graph (Supplemental Figure 11).

8. A. thaliana quantitative disease resistance trait

For the quantitative disease resistance trait (Debieu et al. 2016), the number of markers remaining after quality control using the *k*-merGWAS, SNP analysis, and NodeGWAS was 170,382,079, 793,248 and 10,518,414, respectively; of these, the number of significant associations called by each method was 4,750, 115, and 49,023, respectively. Although all three approaches pinpointed the same 16.7Mb region on chromosome 5, *k*-merGWAS exhibited a high false-positive rate, consistent with its tendencies to over-report (Supplemental Figure 13) - whereas NodeGWAS produced a sharp, well-defined peak capturing only the nodes in LD, without spurious signals.

9. Discussion of NodeGWAS

Unlike *vg call* - which relies on explicit detection and VCF representation of complex structural variants manifesting as nested bubbles (Supplemental Figure 2 and Supplemental Table 2) (Paten et al. 2018, 2017; Garrison et al. 2018, 2022) - NodeGWAS genotypes nodes directly, sidestepping the challenge of SV calling and representation (Romain et al. 2025). Secondly, in contrast to *vg inject*, our method is more efficient and streamlined. *vg inject* requires injecting alignments from BAM files back to the linear reference genome and subsequent separation of SNP and SV discovery. Third, compared to *k*-mer-based GWAS, our method leverages the link field (*L*) in the GFA file to precisely locate variants on the reference genome. *k*-mer-based approaches often over-report loci with substantial redundancy, which are difficult to map and interpret, computationally expensive, and, although seemingly more sensitive, have been shown to significantly increase false positive rates. Fourth, the pangenome can comprehensively capture the genetic diversity of a species and effectively reduce analytical bias caused by reliance on a single reference genome.

Despite its ability to alleviate reference bias through direct graph-based analysis, NodeGWAS is still constrained by several limitations. First, computational scalability remains a challenge for ultra-large polyploid genomes, as the requirements for graph construction, mapping and node-counting increase significantly. Second, highly repetitive sequences may complicate graph topology, potentially leading to non-specific node mapping and reduced statistical power in these regions, though we note that this limitation is shared with linear-reference approaches. Finally, the comprehensiveness of the graph is inherently limited by the quality and representative diversity of the input genomes. Incomplete or biased sampling of founder genomes may reduce sensitivity for rare or population-specific variation. As more high-quality, telomere-to-telomere (T2T) assemblies

are getting integrated into pan-genome frameworks, the resolution and discovery power of NodeGWAS will continue to improve.

1001 Genomes Consortium. Electronic address: magnus.nordborg@gmi.oeaw.ac.at, and 1001 Genomes Consortium. 2016. “1,135 Genomes Reveal the Global Pattern of Polymorphism in Arabidopsis Thaliana.” *Cell* 166 (2): 481–491.

Abney, Mark. 2015. “Permutation Testing in the Presence of Polygenic Variation.” *Genetic Epidemiology* 39 (4): 249–258.

Debieu, Marilyne, Carine Huard-Chauveau, Anne Genissel, Fabrice Roux, and Dominique Roby. 2016. “Quantitative Disease Resistance to the Bacterial Pathogen Xanthomonas Campestris Involves an Arabidopsis Immune Receptor Pair and a Gene of Unknown Function.” *Molecular Plant Pathology* 17 (4): 510–520.

Ebler, Jana, Peter Ebert, Wayne E. Clarke, et al. 2022. “Pangenome-Based Genome Inference Allows Efficient and Accurate Genotyping across a Wide Spectrum of Variant Classes.” *Nature Genetics* 54 (4): 518–525.

Garrison, Erik, Andrea Guarracino, Simon Heumos, et al. 2024. “Building Pangenome Graphs.” *Nature Methods* 21 (11): 2008–2012.

Garrison, Erik, Zev N. Kronenberg, Eric T. Dawson, Brent S. Pedersen, and Pjotr Prins. 2022. “A Spectrum of Free Software Tools for Processing the VCF Variant Call Format: Vcflib, Bio-Vcf, cyvcf2, Hts-Nim and Slivar.” *PLoS Computational Biology* 18 (5): e1009123.

Garrison, Erik, Jouni Sirén, Adam M. Novak, et al. 2018. “Variation Graph Toolkit Improves Read Mapping by Representing Genetic Variation in the Reference.” *Nature Biotechnology* 36 (9): 875–879.

Healey, A. L., O. Garsmeur, J. T. Lovell, et al. 2024. “The Complex Polyploid Genome Architecture of Sugarcane.” Nature 628, 804–810.

He, Cheng, Jacob D. Washburn, Nathaniel Schleif, et al. 2024. “Trait Association and Prediction through Integrative K-Mer Analysis.” *The Plant Journal: For Cell and Molecular Biology* 120 (2): 833–850.

Hickey, Glenn, David Heller, Jean Monlong, et al. 2020. “Genotyping Structural Variants in Pangenome Graphs Using the vg Toolkit.” *Genome Biology* 21 (1): 35.

Hickey, Glenn, Jean Monlong, Jana Ebler, et al. 2024. “Pangenome Graph Construction from Genome Alignments with Minigraph-Cactus.” *Nature Biotechnology* 42 (4): 663–673.

Huang, Yumin, Yixing Zhang, Qing Zhang, et al. 2026. “Multiscale Pangenome Graphs Empower the Genomic Dissection of Mixed-Ploidy Sugarcane Species.” *Science (New York, N.Y.)* 391 (6785): eadx1616.

Kang, Hyun Min, Jae Hoon Sul, Susan K. Service, et al. 2010. “Variance Component Model to Account for Sample Structure in Genome-Wide Association Studies.” *Nature Genetics* 42 (4): 348–354.

Kang, Hyun Min, Noah A. Zaitlen, Claire M. Wade, et al. 2008. “Efficient Control of Population Structure in Model Organism Association Mapping.” *Genetics* 178 (3): 1709–1723.

Kang, Minghui, Haolin Wu, Huanhuan Liu, et al. 2023. “The Pan-Genome and Local Adaptation of Arabidopsis Thaliana.” *Nature Communications* 14 (1): 6259.

Karikari, Benjamin, Marc-André Lemay, and François Belzile. 2023. “K-Mer-Based Genome-Wide Association Studies in Plants: Advances, Challenges, and Perspectives.” *Genes* 14 (7): 1439.

Lian, Qichao, Bruno Huettel, Birgit Walkemeier, et al. 2024. “A Pan-Genome of 69 Arabidopsis Thaliana Accessions Reveals a Conserved Genome Structure throughout the Global Species Range.” *Nature Genetics* 56 (5): 982–991.

Naish, Matthew, Michael Alonge, Piotr Wlodzimierz, et al. 2021. “The Genetic and Epigenetic Landscape of the Arabidopsis Centromeres.” *Science (New York, N.Y.)* 374 (6569): eabi7489.

Parmigiani, Luca, Erik Garrison, Jens Stoye, Tobias Marschall, and Daniel Doerr. 2024. “Panacus: Fast and Exact Pangenome Growth and Core Size Estimation.” *Bioinformatics (Oxford, England)* 40 (12): btae720.

Paten, Benedict, Jordan M. Eizenga, Yohei M. Rosen, Adam M. Novak, Erik Garrison, and Glenn Hickey. 2018. “Superbubbles, Ultrabubbles, and Cacti.” *Journal of Computational Biology: A Journal of Computational Molecular Cell Biology* 25 (7): 649–663.

Paten, Benedict, Adam M. Novak, Jordan M. Eizenga, and Erik Garrison. 2017. “Genome Graphs and the Evolution of Genome Inference.” *Genome Research* 27 (5): 665–676.

Quinlan, Aaron R., and Ira M. Hall. 2010. “BEDTools: A Flexible Suite of Utilities for Comparing Genomic Features.” *Bioinformatics (Oxford, England)* 26 (6): 841–842.

Romain, Sandra, Siegfried Dubois, Fabrice Legeai, and Claire Lemaitre. 2025. “Investigating the Topological Motifs of Inversions in Pangenome Graphs.” In *bioRxiv*. March 17. https://doi.org/10.1101/2025.03.14.643331.

Sirén, Jouni, Jean Monlong, Xian Chang, et al. 2021. “Pangenomics Enables Genotyping of Known Structural Variants in 5202 Diverse Genomes.” *Science* 374 (6574): abg8871.

Voichek, Yoav, and Detlef Weigel. 2020. “Identifying Genetic Variants Underlying Phenotypic Variation in Plants without Complete Genomes.” *Nature Genetics* 52 (5): 534–540.

Wang, Tianyou, Baiyu Wang, Xiuting Hua, et al. 2023. “A Complete Gap-Free Diploid Genome in Saccharum Complex and the Genomic Footprints of Evolution in the Highly Polyploid Saccharum Genus.” *Nature Plants* 9 (4): 554–571.

Zhang, Feng, Li Qu, Yincong Gu, Zhi-Hong Xu, and Hong-Wei Xue. 2022. “Resequencing and Genome-Wide Association Studies of Autotetraploid Potato.” *Molecular Horticulture* 2 (1): 6.

Zhang, Jisen, Yiying Qi, Xiuting Hua, et al. 2025. “The Highly Allo-Autopolyploid Modern Sugarcane Genome and Very Recent Allopolyploidization in Saccharum.” *Nature Genetics* 57 (1): 242–253.

Zhang, Jisen, Xingtan Zhang, Haibao Tang, et al. 2018. “Allele-Defined Genome of the Autopolyploid Sugarcane Saccharum Spontaneum L.” *Nature Genetics* 50 (11): 1565–1573.

Zhang, Qing, Yiying Qi, Haoran Pan, et al. 2022. “Genomic Insights into the Recent Chromosome Reduction of Autopolyploid Sugarcane Saccharum Spontaneum.” *Nature Genetics* 54 (6): 885–896.

Zhou, Xiang, and Matthew Stephens. 2012. “Genome-Wide Efficient Mixed-Model Analysis for Association Studies.” *Nature Genetics* 44 (7): 821–824.

Supplementary Figure

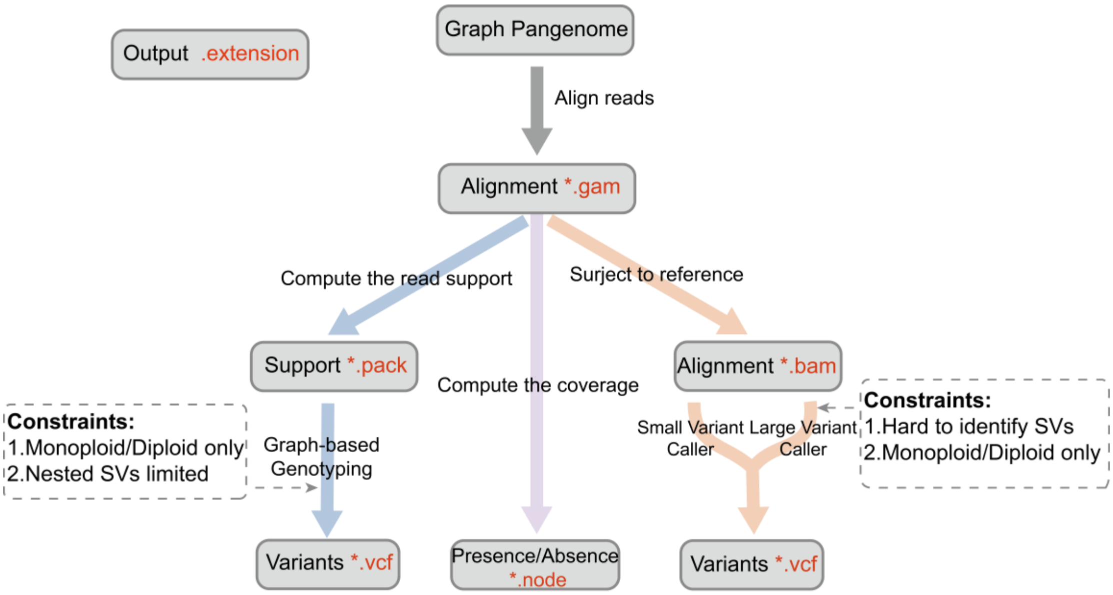

Supplemental Figure 1. Schematic of the GWAS workflow based on a graph pangenome. The workflow utilizes a graph pangenome as the reference. Following read alignment, an alignment file in .gam format is generated, which is then processed through three distinct analytical pathways: Left pathway (Graph-based genotyping): Genotyping is performed by computing read support (outputting .pack files) to ultimately generate variants. The constraints of this path include limited support for non-monoploid/diploid genomes and restricted capability in handling nested SVs. Middle pathway (Node-based analysis): This path directly computes the coverage of graph nodes to analyze Node presence/absence variations. Right pathway: Graph alignments are projected back onto a linear reference genome and converted into standard .bam files. Subsequently, small or large variant callers are employed to generate .vcf files. This approach faces challenges in de novo identifying SVs and is similarly constrained by organism polyploidy (supporting monoploid/diploid only).

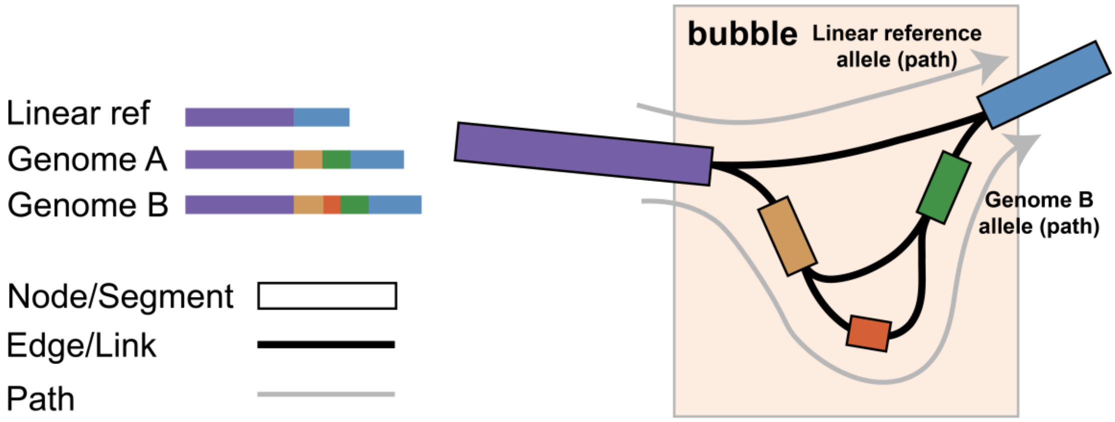

Supplemental Figure 2. Representation of a polymorphic locus in a pangenome graph takes the form of a ‘‘bubble’’ structure, which diverges at a source node where genomic sequences differ and converges at a sink node where sequences realign. Each distinct path from the source to the sink represents a specific allele or haplotype.

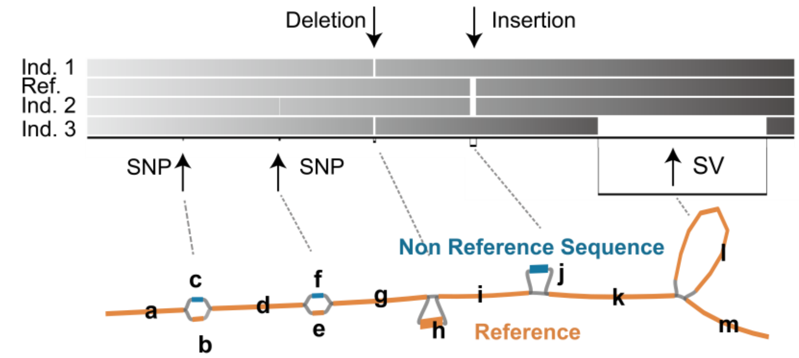

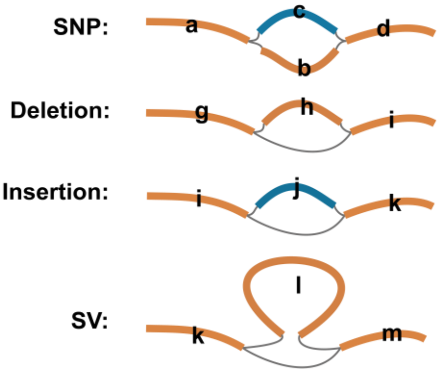

	Ind.1	Ind.2	Ind.3
a	1	1	1
b	1	1	0
c	0	0	1
d	1	1	1
e	1	1	0
f	0	0	1
g	1	1	1
h	0	1	0
i	1	1	1
j	1	0	1
k	1	1	1
l	1	1	0
m	1	1	1

Supplemental Figure 3. Illustration of the node-based variation representation in NodeGWAS. Four individual sequences (Ind. 1-3 and a Reference) containing two SNPs, one deletion, one insertion, and one structural variant (SV). Node “*h*” specifically captures a deletion event, while node “*l*” represents a large SV sequence. All variations within the pangenome can be represented by distinct nodes. By constructing a nodetable, we can effectively reconstruct the node-level information.

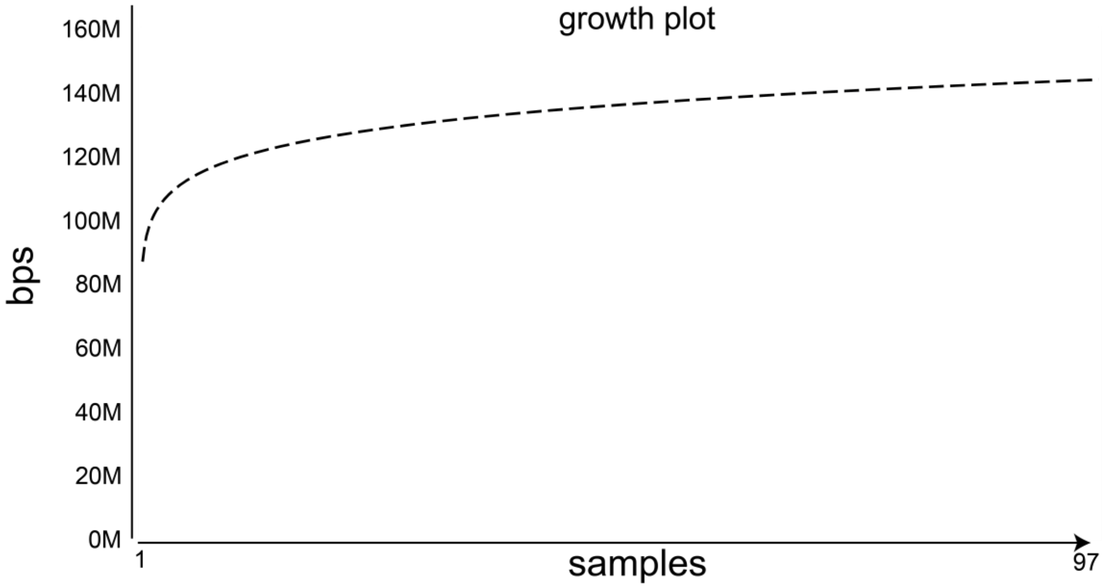

Supplemental Figure 4. Pangenome growth curves for 97 *A. thaliana*.

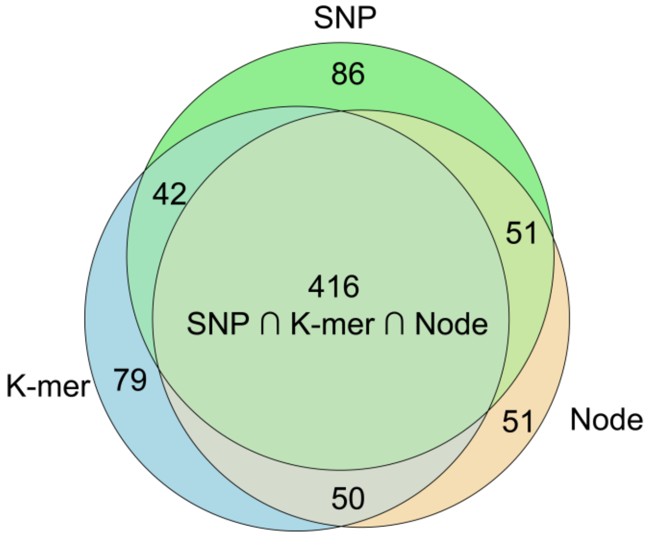

Supplemental Figure 5. Overlap between phenotypic traits and the hits derived from SNP, *k*-mer, and Node.

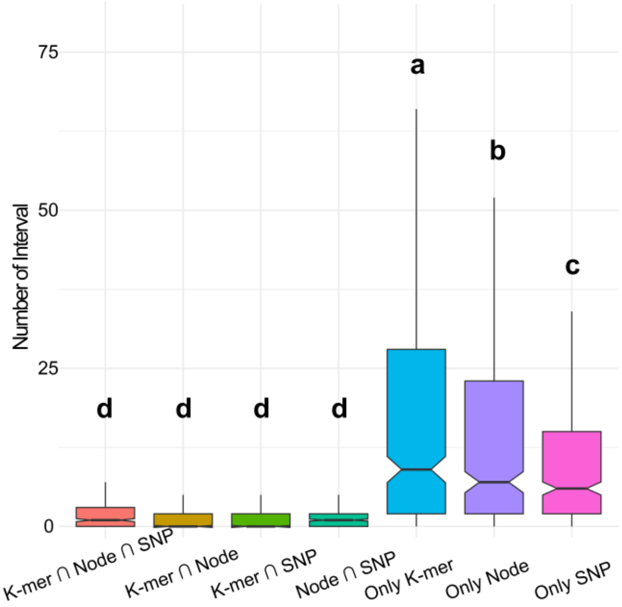

Supplemental Figure 6. Boxplot of the number of intervals in the intersection and union of three methods. Differences among groups were assessed using one-way ANOVA followed by LSD multiple comparison tests with Benjamini-Hochberg adjustment. Different letters indicate significant differences at $P < 0.05$.

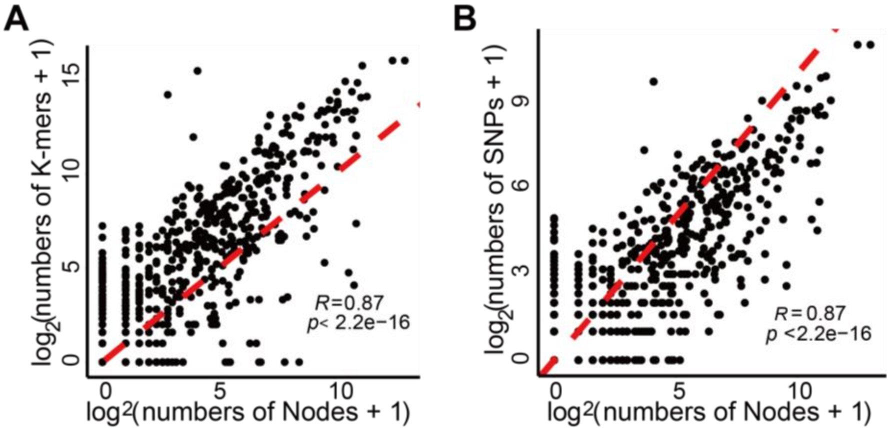

Supplemental Figure 7. Correlation between the number of significant markers. A: Correlation between the number of significant nodes and *k*-mers. B: Correlation between the number of significant nodes and SNPs. Pearson correlation coefficient (*R*) was calculated, and its significance (*P* values) was tested using t-tests.

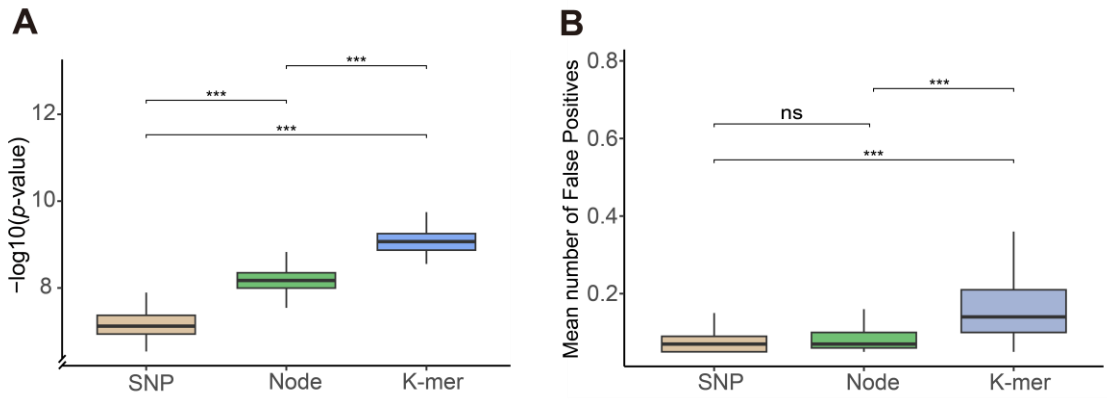

Supplemental Figure 8. Performance comparison of SNP, Node, and *k*-mer methods.

(A) Comparison of statistical significance. The y-axis represents -log10(*P*), with higher values indicating higher error rate. (B) Comparison of The Mean number of False Positives (MFP). The y-axis represents the MFP. Significance was calculated using the Wilcoxon test, "ns" indicates no significant difference, *** indicates $P \leq 0.001$.

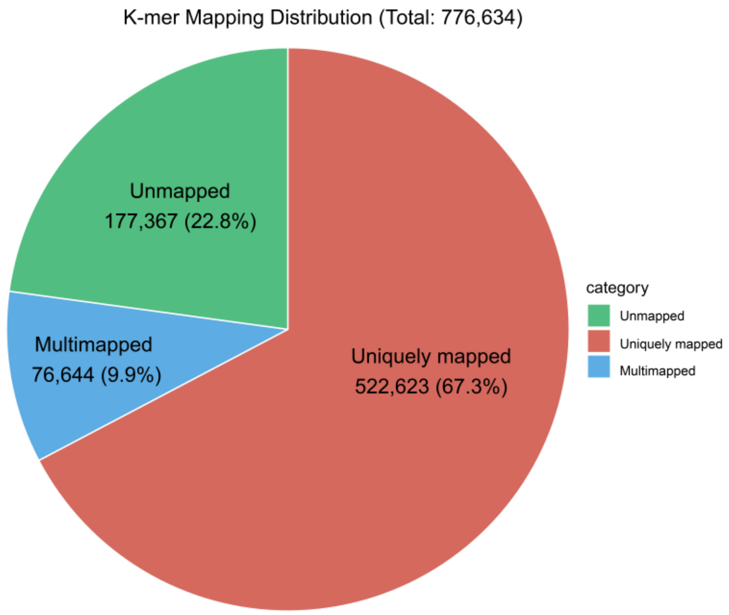

Supplemental Figure 9. The distribution of *k*-mer Mapping.

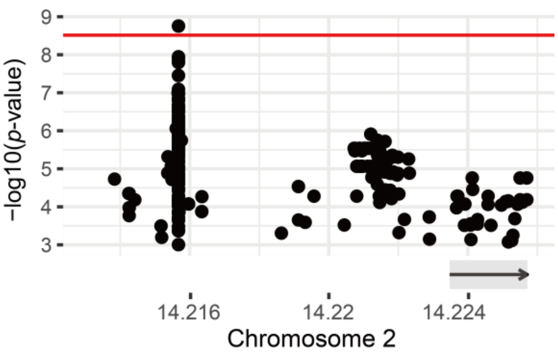

Supplemental Figure 10. Manhattan plot of a zoomed-in candidate region on chromosome 2, showing several significant loci located upstream of *AT2G27380*.

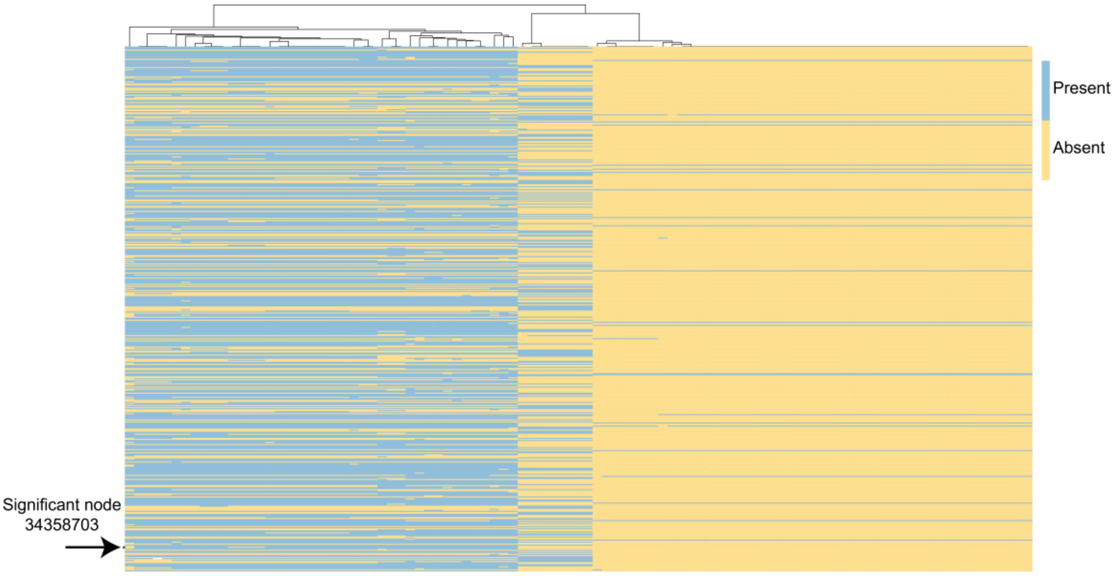

Supplemental Figure 11. Node-level presence/absence variation around the *EPR1* locus. Rows represent distinct nodes, while columns correspond to individual samples. The color scale denotes the node status: light blue represents presence, and light yellow represents absence. The arrow indicates a significant node identified by NodeGWAS.

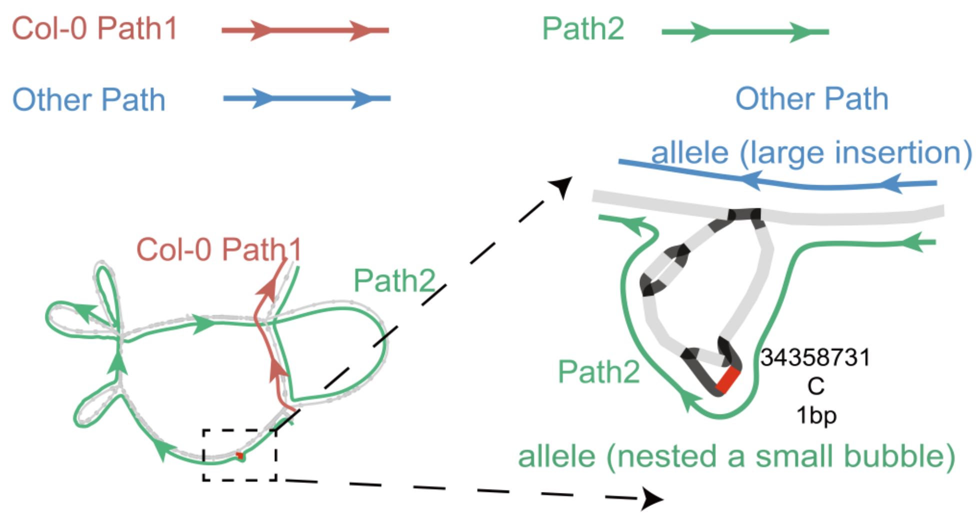

Supplemental Figure 12. Graph pangenome view of the associated locus (node ID: 34258731, sequence: C, length: 1 bp, highlighted in red). Left, overview of the local graph with alternative haplotype paths. Right, zoomed inset showing the red node as a single-base variant nested within a larger insertion (blue path). Arrows and colored paths indicate distinct haplotypes.

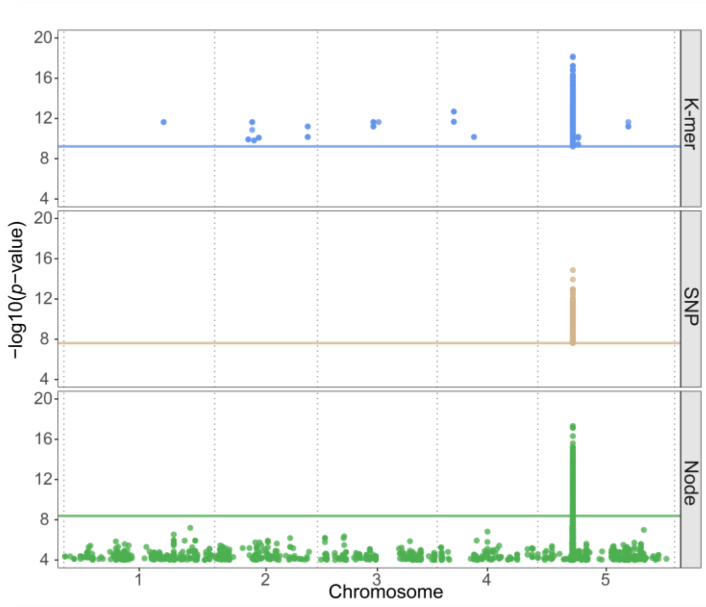

Supplemental Figure 13. Manhattan plots of quantitative disease resistance, with different colors representing different methods. Horizontal dashed lines indicate the genome-wide significance threshold used for each method. For the *k*-mer and SNP methods, only significant loci are displayed because the original *k*-merGWAS study only provided significant sites.

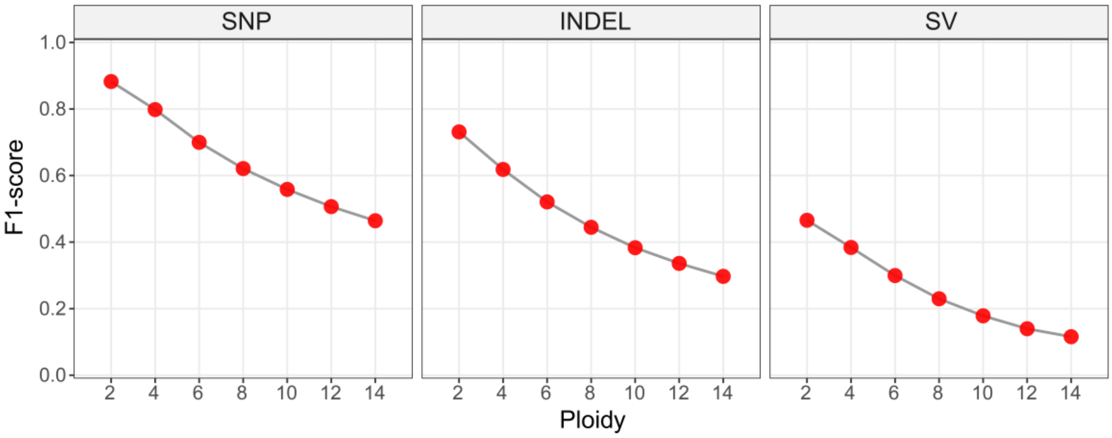

Supplemental Figure 14. F1-scores for variant detection across varying ploidy levels of *A. thaliana*, based on simulated sequencing data and a linear genome reference.

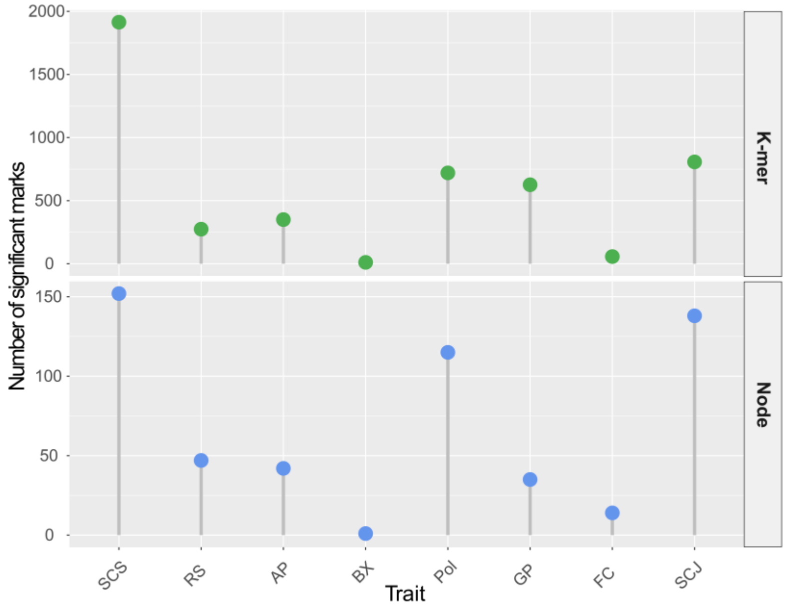

Supplemental Figure 15. Number of significant marks identified by SNP-, Node-, and *k*-mer-based methods. Sucrose-related traits. SCS: sucrose content in sugarcane; RS: reducing sugars; AP: apparent purity; BX: Brix; Pol: polarization; GP: gravity purity; FC: fiber content; SCJ: sucrose content in juice.

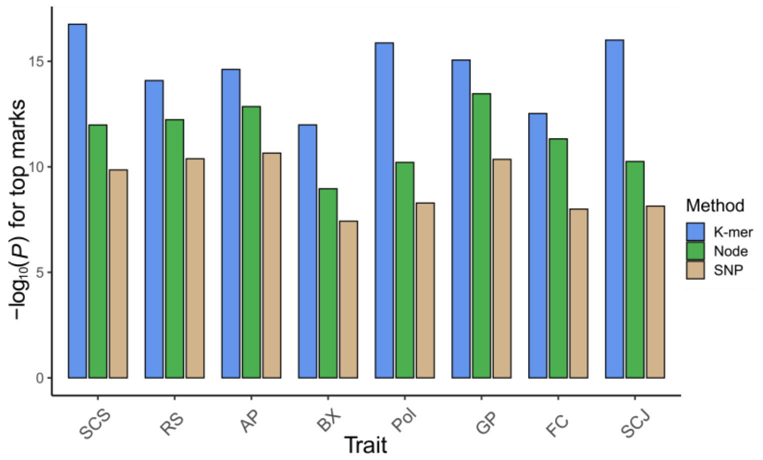

Supplemental Figure 16. *P* values of top marks identified by SNP-, Node-, and *k*-mer-based methods. Sucrose-related traits. SCS: sucrose content in sugarcane; RS: reducing sugars; AP: apparent purity; BX: Brix; Pol: polarization; GP: gravity purity; FC: fiber content; SCJ: sucrose content in juice.

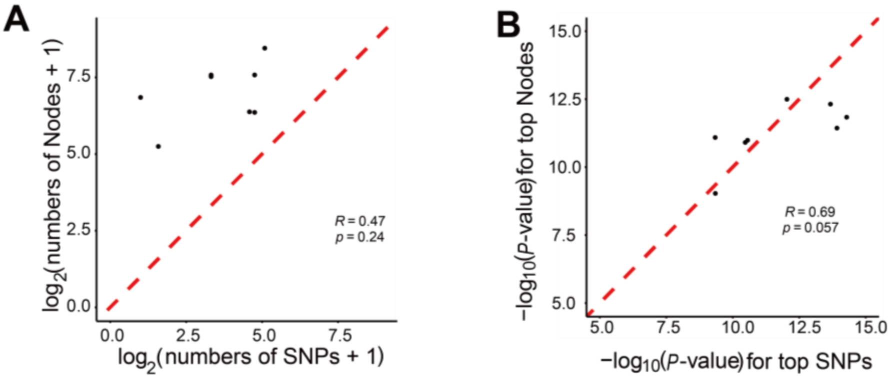

Supplemental Figure 17. Comparison of significant Nodes and significant SNPs.

A: Correlation between the number of significant Nodes and SNPs in sugarcane. B: Correlation of *P* values of top Nodes with SNPs in sugarcane. Pearson correlation coefficient (*R*) was calculated, and its significance (*P* value) was tested using t-tests.

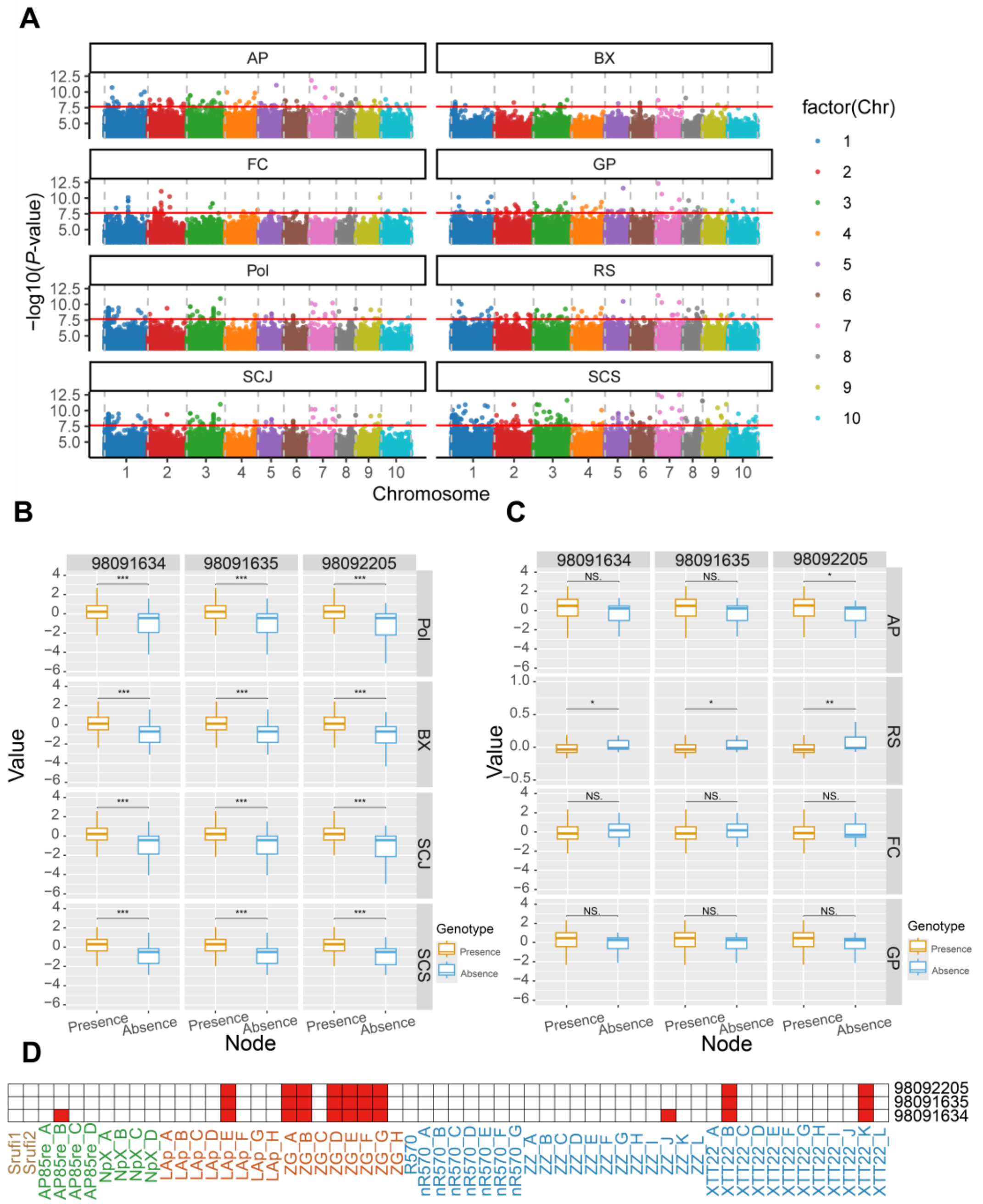

Supplemental Figure 18. GWAS identification of candidate loci for sucrose-related traits

A: Manhattan plot results for the NodeGWAS on 8 sugar-related traits. SCS: sucrose content in sugarcane; SCJ: sucrose content in juice; RS: reducing sugars; AP: apparent purity; GP: gravity purity; BX: Brix; Pol: polarization; FC: fiber content. **B-C:** Boxplot plot for 8 sucrose-related traits of three candidate nodes of *Erufi.06G007100*. significance (*P* value) was tested using t-tests, "NA" indicates no significant difference, *** indicates $P \leq 0.001$. **D:** The sources of three candidate nodes of *Erufi.06G007100*. The red block represents the node present in the haplotype.

Supplementary Table

Supplemental Table 1. Previous studies using *k*-merGWAS.

Species	Number of traits	Using *k*-mers Only	Reference
Arabidopsis, tomato, maize	2,000	YES	(Voichek and Weigel 2020)
wheat	2	NO	(Kale et al. 2022)
soybean	13	NO	(Lemay et al. 2023)
maize	4	YES	(He et al. 2024)
wheat	1	YES	(Zhang et al. 2024)
Lactuca sativa	1	YES	(Selvanayagam et al. 2025)

Supplemental Table 2. Definitions of terms in the graph pangenome used in this study.

Term	Definition
Node/Segment	A segment of DNA that is shared or unique among individuals. Depending on graph construction, nodes may be short (e.g., variant-scale) or represent longer haplotype segments.
Link/Edge	A connection linking two nodes (or segments) that are adjacent in one or more genomes. When the overlap is 0-bp, the B segment follows directly after A.
Path	A path is defined as an ordered sequence of nodes connected by edges. In a full pangenome graph, a path generally represents the complete chromosome of a specific individual. When restricted to a subgraph, the path represents a localized sequence segment. The biological interpretation of a path is an allele or haplotype.
Bubble (simple)	A bubble is a subgraph defined by two nodes, A and B, between which there exist at least two distinct paths. The different paths within the bubble represent alternative alleles at that locus. A bubble can encode simple variations, including SNPs, Indels, and SVs.
Nested bubble	Nested bubbles representing complex structural variations, where smaller variants are embedded within larger ones
Surject	Surject describes a computational projection of sequence alignments from a graph pangenome onto a linear reference genome, thereby translating graph-specific alignment formats (GAM/GAF) into standard linear alignment file formats (SAM/BAM).

Supplemental Table 3. Number of QTLs identified by *k*-merGWAS and NodeGWAS and their overlap.

Trait	*k*-mer	Node	Overlapped
SCS	173	38	9
RS	21	40	0
AP	36	1	1
BX	1	0	0
Pol	14	2	2
GP	41	30	1
FC	10	7	0
SCJ	56	26	2

Supplemental Table 4. Computing run time and memory usage of NodeGWAS and GATK.

GATK Pipeline	Real time (min)	CPU time (min)	Peak RAM (GB)	Threads
Read Mapping (BWA)	290.71	2,585.25	3.54	10
Coordinate Sorting	4.32	19.41	8.39	10
Mark Duplicates	20.67	28.86	30.99	10
Variant Calling	2,733.01	9,643.40	12.73	10
GenotypeGVCFs	65.41	65.24	1.19	10
Total time in one sample	3,114.12	12,342.16		
**NodeGWAS Pipeline**	**Real time (min)**	**CPU time (min)**	**Peak RAM (GB)**	**Threads**
Read Mapping (VG giraffe)	304.61	3,022.79	26.41	10
Coverage	67.7	185.57	26.79	10
Total time in one sample	372.31	3,208.36		
